# Design, Synthesis, and Biological Evaluation of 3-Substituted-Indolin-2-One Derivatives as Potent Anti-Inflammatory Agents

**DOI:** 10.3390/ijms24032066

**Published:** 2023-01-20

**Authors:** Sung Jin Kim, Sang Hyuk Lee, Heesu Lee, Myoung-Sook Shin, Jae Wook Lee

**Affiliations:** 1 College of Korean Medicine, Gachon University, Seongnam-si 13120, Republic of Korea; 2 Natural Product Research Center, Korea Institute of Science and Technology (KIST), Gangneung-si 25451, Republic of Korea; 3 Department of Anatomy, College of Dentistry, Gangneung Wonju National University (GWNU), Gangneung-si 25457, Republic of Korea

**Keywords:** 3-substituted-indolin-2-one derivatives, inflammation, macrophages, cytokines

## Abstract

This study aimed to synthesize and evaluate the anti-inflammatory activity of 3-substituted-indolin-2-one derivatives. Cell viability of 3-substituted-indolin-2-one derivatives was measured with the EZ-Cytox reagent; interleukin (IL)-6, tumor necrosis factor (TNF)-α, and inducible NOS mRNA levels were measured using Taqman qRT-PCR; pro-inflammatory cytokine IL-6 and TNF-α levels were determined using ELISA kits; the phosphorylation of Akt, JNK, ERK, p38, p65, and IκB protein levels were measured by immunoblotting. Among the nineteen 3-substituted-indolin-2-one derivatives synthesized, 3-(3-hydroxyphenyl)-indolin-2-one showed the highest anti-inflammatory activity, inhibiting the nitric oxide production related to inflammation, suppressing the production of TNF-α and IL-6 in a concentration-dependent manner and mRNA expression. Moreover, 3-(3-hydroxyphenyl)-indolin-2-one significantly inhibited lipopolysaccharide (LPS)-induced signal pathways such as the Akt, MAPK, and NF-κB signaling pathways. Our findings revealed that a 3-substituted-indolin-2-one derivative, 3-(3-hydroxyphenyl)-indolin-2-one, possesses excellent anti-inflammatory activity and can be considered for future research.

## 1. Introduction

Inflammation is a physiological and pathological response that activates immune and non-immune cells that protect host cells or organs from infectious species, including bacteria, viruses, and toxins, by eliminating pathogens and promoting tissue repair and recovery [1,2,3]. In well-controlled inflammation, the initiation phase is involved in host defense by rapid and robust immune activation. The resolution phase curtails inflammation and restores tissue homeostasis when the danger signal is eliminated [4]. Uncontrolled inflammation can cause chronic inflammatory diseases, such as arthritis, colitis, and asthma. These diseases are associated with severe tissue damage and an increased risk for the development of cardiovascular diseases, cancer, and osteoporosis [5,6,7,8].

The inflammatory response involves various cells, including neutrophils, macrophages, and lymphocytes [9]. In macrophages, inflammation is initiated by lipopolysaccharide (LPS) which is a lipid and a polysaccharide of Gram-negative bacteria. LPS can increase the expression of tumor necrosis factor α (TNF-α) and the release of inflammatory cytokines such as interleukin (IL)-6, IL-1, IL-1β, and nitric oxide (NO) [10,11]. As a result, LPS can induce an acute inflammatory response. Activated macrophages enhance the expression of cyclooxygenase-2 (COX-2) and inducible nitric oxide synthase (iNOS), which act as inflammatory mediators and immune modulators.

**Figure 1:**
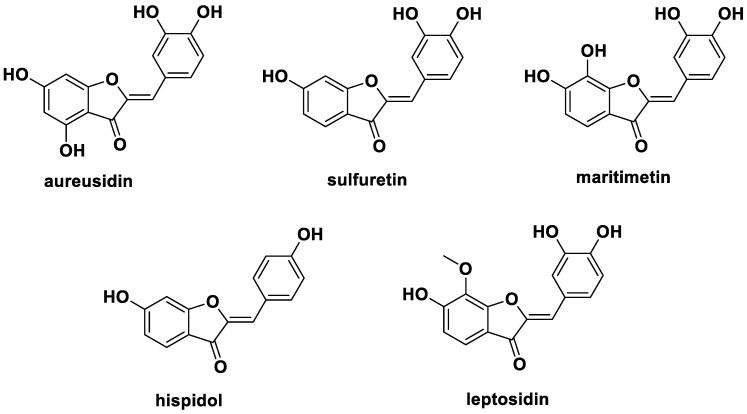
Structures of aurones isolated from plants.

Aurones and 2-benzylidenebenzofuran-3(2H)-ones are isomeric structures of flavones and are naturally occurring yellow pigments isolated from plants such as vegetables and flowers (Figure 1) [12,13]. Natural aurones can be obtained from the biosynthesis of chalcones by the key enzyme auresidin synthase [14], as well by a typical biosynthetic procedure from other flavonoids [15]. Representative naturally occurring aurones include aureusidin [16], sulfuretin [17,18,19], and maritimetin [20,21].

Aurones have been reported to have anticancer [22,23], antiparasitic [24], antileishmanial [25,26], antibacterial [27,28], and antifungal [29,30] properties. In a recent report, aurones also showed potent anti-inflammatory activities with 74–100% inhibition of both TNF-α and IL-6 at 10 μM aurone derivatives in human monocytes [31].

The structure of the aurones consists of benzofuran-3-one and benzylidene, which are attached to two positions on benzofuran-3-one. Previous studies synthesized azaaurones as aurone derivatives and evaluated their biological effects [32,33,34,35]. In addition, an azaaurone scaffold has been utilized as an antimalarial agent [36].

**Figure 2:**
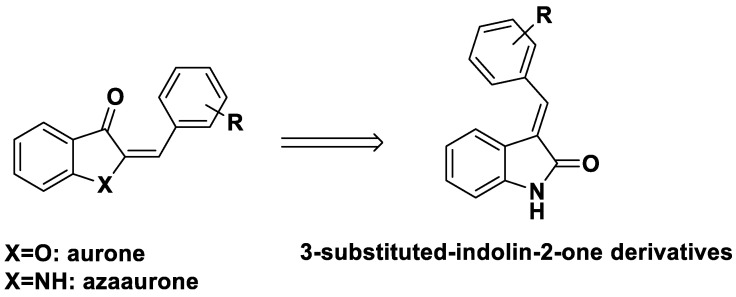
Rational design of 3-substituted-indolin-2-one derivatives.

To identify structurally similar aurones with anti-inflammatory activity, we designed 3-substituted-indolin-2-one derivatives based on the aurone and azaaurone structures (Figure 2). The nineteen designed 3-substituted-indolin-2-one derivatives were synthesized and assayed for their anti-inflammatory activities.

**Figure 3:**
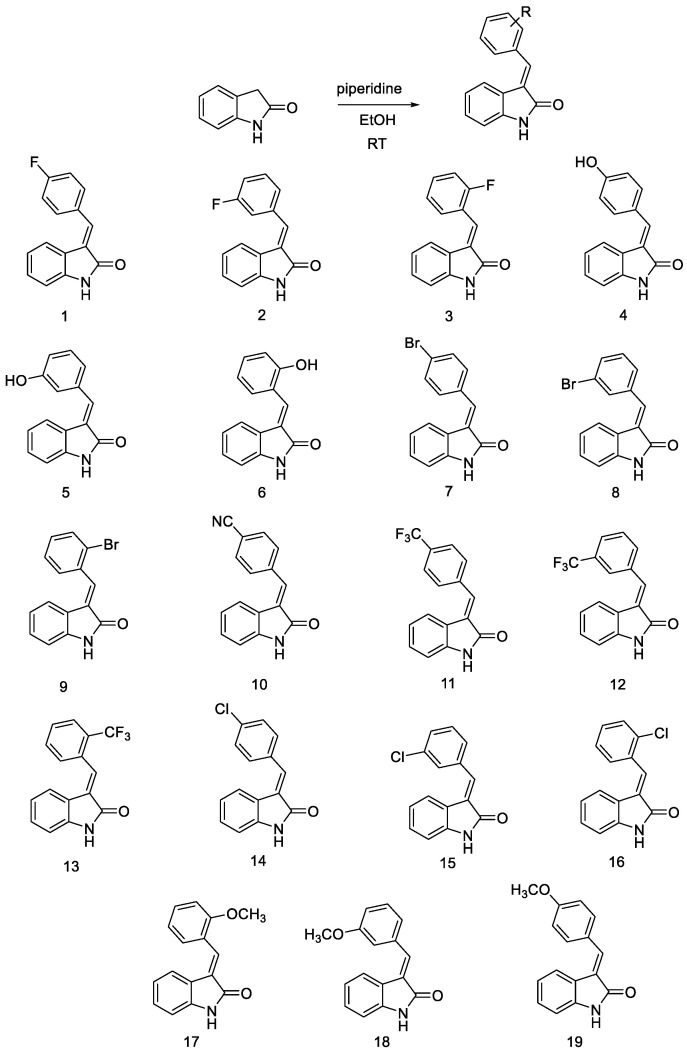
Synthetic scheme and structures of 3-substituted-indolin-2-one derivatives.

The compounds described in this study were prepared using straightforward chemical synthesis (Figure 3). The nineteen derivatives were synthesized following a previously described method with minor modifications [37,38]. The reaction mixtures were purified by preparative high-performance liquid chromatography to afford major compounds. The desired derivatives were characterized by ^1^H nuclear magnetic resonance (NMR), ^13^C NMR, and liquid chromatography–mass spectrometry (Supplementary Figure S1). In the previous report, chemical shifts of H-2′ and H-6′ protons in the phenyl ring at the C-3 position of 3-substituted-indolin-2-one were observed to determine *E* and *Z* isomers. We followed the reported method to determine the configurations of nineteen synthesized analogs [37,38].

The synthesized analogs were assayed for their biological activities, such as anti-inflammatory activity including production of nitric oxide, TNF-α, IL-6, etc., using RAW264.7 murine macrophages. Among the nineteen 3-substituted-indolin-2-one derivatives, 3-(3-hydroxyphenyl)-indolin-2-one showed the highest anti-inflammatory activity. Therefore, we elucidated its underlying molecular signaling pathways using RAW264.7 cells.

## 2. Results

### 2.1. Effects of 3-Substituted-Indolin-2-One Derivatives on Nitric Oxide Secretion in LPS-Stimulated RAW264.7 Cells

**Figure 4:**
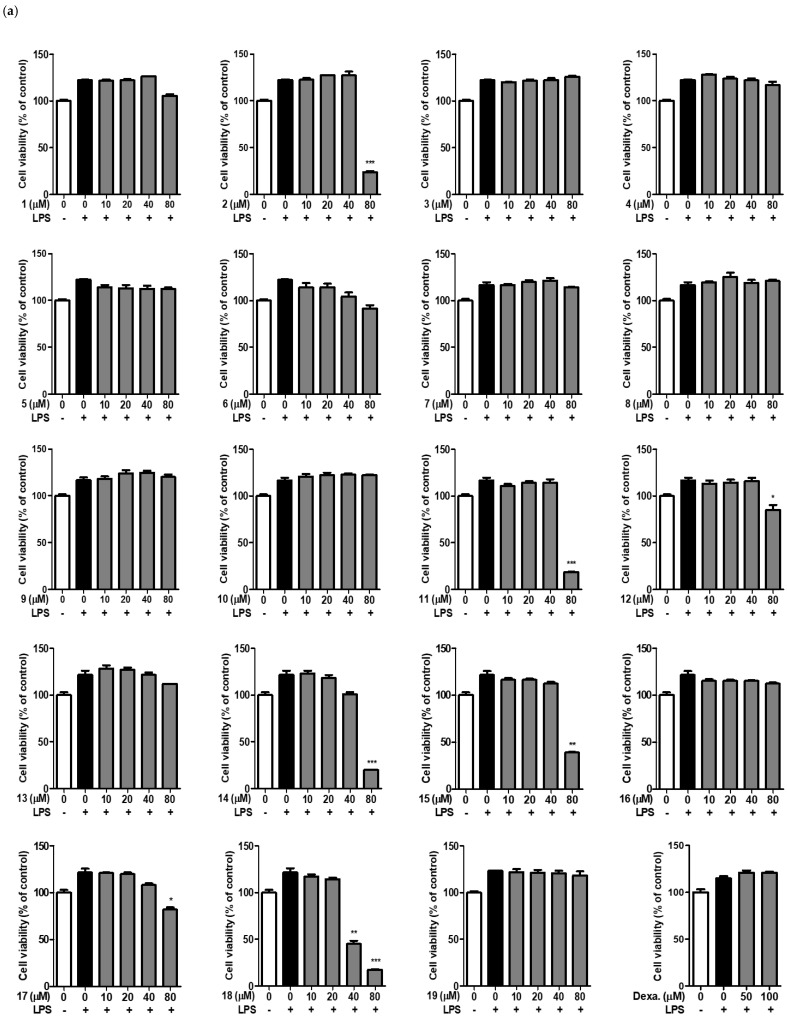

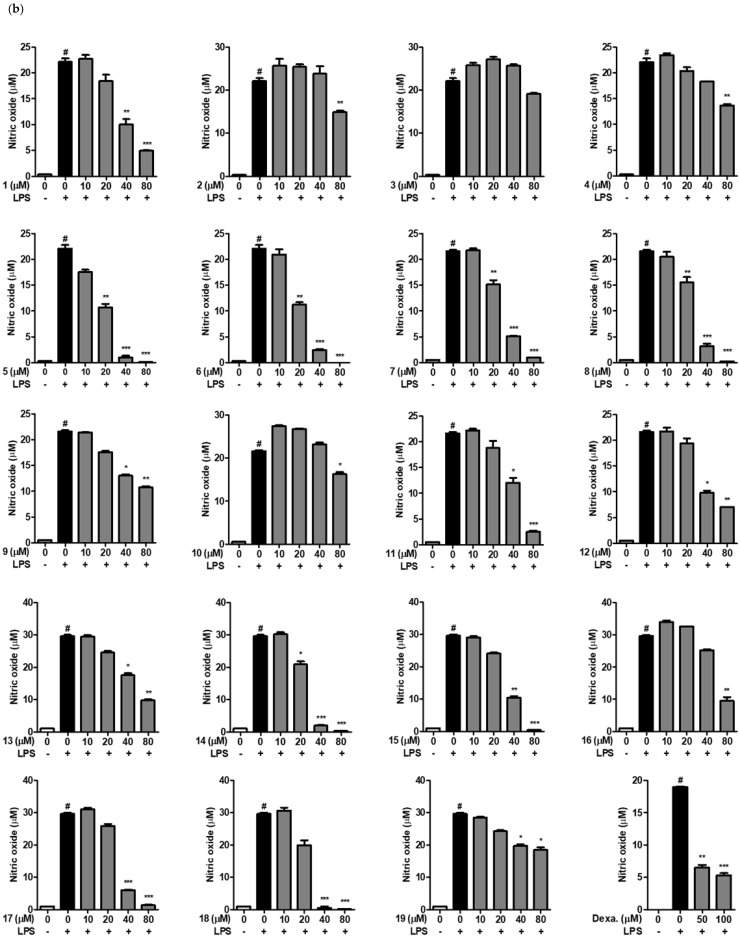
Effects of nineteen 3-substituted-indolin-2-one derivatives on cytotoxicity and nitric oxide production in lipopolysaccharide (LPS)-stimulated RAW264.7 cells; (-): non-treated group, (+): LPS-treated group. (**a**) RAW264.7 cells (1 × 10^5^/well, 96-well plates) treated with nineteen 3-substituted-indolin-2-one derivatives for 2 h and incubated with LPS for 18 h. Cytotoxicity measured by EZ-cytox cell viability solution as described in the Materials and Methods section; (**b**) RAW264.7 cells (1 × 10^5^/well, 96-well plates) treated with 3-substituted-indolin-2-one derivatives at varying concentrations (10, 20, 40, and 80 µM) for 2 h, and then stimulated with LPS (500 ng/mL) for 18 h. Cell supernatants were collected, and nitric oxide concentration was determined using Griess reagents as described in the Materials and Methods. White bar: non-treated group, black bar: LPS treated group, gray bar: sample treated group. ^#^ *p* < 0.0001 vs. the control group. *** *p* < 0.0001, ** *p* < 0.001 or * *p* < 0.05 vs. the LPS group.

To analyze the anti-inflammatory activity of 3-substituted-indolin-2-one derivatives, we first determined their cytotoxicity using the murine macrophage cell line RAW264.7. RAW264.7 cells were treated with 3-substituted-indolin-2-one derivatives at concentrations of 10, 20, 40, and 80 μM for 2 h and then treated with LPS at 500 ng/mL for 20 h. As shown in Figure 4a, 3-substituted-indolin-2-one derivatives such as 1, 3, 4, 5, 6, 7, 8, 9, 10, 13, and 16 did not show cytotoxicity at any of the treated concentrations (10–80 μM). However, several derivatives, such as 2, 11, 12, 14, 15, 17, and 18, showed cytotoxicity at 80 μM. 

Next, we investigated which 3-substituted-indolin-2-one derivatives displayed strong nitric oxide inhibition in LPS-stimulated RAW264.7 cells. As shown in Figure 4b, 3-substituted-indolin-2-one 5, 6, 7, and 8 significantly suppressed nitric oxide secretion in a concentration-dependent manner.

In general, electronegative groups (e.g., Cl, Br, and CF_3_) that substituted derivatives showed a 50% suppression effect on nitric oxide secretion of about 40 μM. Interestingly, ortho and meta OH-substituted derivatives 5 and 6 showed 50% suppression of NO secretion from 20 μM. Furthermore, 3-(3-hydroxyphenyl)-indolin-2-one showed stronger suppression of LPS-induced nitric oxide production than dexamethasone, a non-steroidal anti-inflammatory drug (NSAID), and a positive control of the anti-inflammatory agent, without cytotoxicity to RAW264.7 cells. Therefore, we investigated 3-(3-hydroxyphenyl)-indolin-2-one to elucidate the molecular mechanisms underlying its anti-inflammatory activity.

### 2.2. Effect of 3-(3-Hydroxyphenyl)-Indolin-2-One on IL-6 and TNF-α Production in RAW264.7 Cells

**Figure 5:**
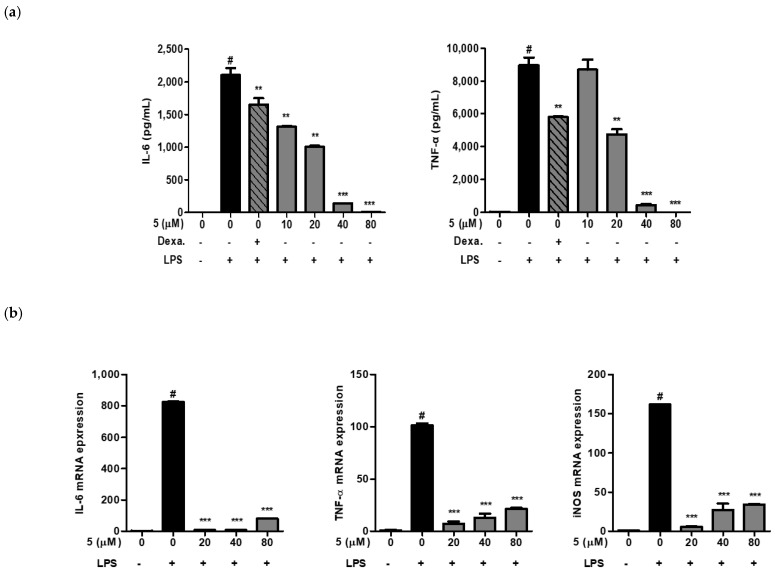
Effects of 3-(3-hydroxyphenyl)-indolin-2-one on interleukin-6 (IL-6), tumor necrosis factor-α (TNF-α) production, and inducible nitric oxide synthase (iNOS) mRNA expression in lipopolysaccharide (LPS)-stimulated RAW264.7 cells. (**a**) RAW264.7 cells (1 × 10^5^/well, 96-well plates) treated with 3-(3-hydroxyphenyl)-indolin-2-one at varying concentrations (10, 20, 40, and 80 µM) for 2 h and then stimulated with LPS (500 ng/mL) for 18 h. Cell supernatant was collected and then analyzed using an enzyme-linked immunosorbent assay kit for cytokines (IL-6 and TNF-α). (**b**) RAW264.7 macrophages (2.0 × 10^6^ cells/6-cm dish) were treated with varying concentrations of 3-(3-hydroxyphenyl)-indolin-2-one (20, 40 and 80 µM) for 2 h, and then stimulated with LPS (500 ng/mL) for 6 h. IL-6, TNF-α, and iNOS mRNA expression levels were measured by real-time quantitative reverse transcription-polymerase chain reaction (qRT-PCR). White bar: non-treated group, black bar: LPS treated group, gray bar: sample treated group. ^#^ *p* < 0.0001 vs. the control group. *** *p* < 0.0001 or ** *p* < 0.001 vs. the LPS group.

Macrophages produce inflammatory mediators, such as TNF-α, nitric oxide, and IL-6. Bacterial endotoxins and inflammatory stimuli induce TNF-α, nitric oxide, and IL-6 mRNA expression in macrophages. We next investigated whether 3-(3-hydroxyphenyl)-indolin-2-one inhibited TNF-α and IL-6 cytokine production as well as mRNA expression in LPS-induced RAW264.7 cells. As shown in Figure 5, pretreatment with 3-(3-hydroxyphenyl)-indolin-2-one significantly inhibited LPS-induced TNF-α and IL-6 production in a concentration-dependent manner (Figure 5a). Similarly, we confirmed the inhibition of TNF-α and IL-6 mRNA expression by 3-(3-hydroxyphenyl)-indolin-2-one treatment in Figure 5b. In addition, iNOS mRNA was inhibited by 3-(3-hydroxyphenyl)-indolin-2-one treatment, which correlated with the nitric oxide inhibition by 3-(3-hydroxyphenyl)-indolin-2-one in Figure 4b. Taken together, these results indicate that 3-(3-hydroxyphenyl)-indolin-2-one might possess anti-inflammatory activity in LPS-induced RAW264.7 cells.

### 2.3. Effect of 3-(3-Hydroxyphenyl)-Indolin-2-One on iNOS Expression in LPS-Induced RAW264.7 Cells

NO is an inorganic vitreous substance produced by nitric oxide synthase and is involved in various biological processes such as the immune response, cytotoxicity, and neurotransmission. NO is produced from l-arginine by nitric oxide synthases (NOS), neuronal NOS, endothelial NOS, and iNOS present in various tissues and cells.

**Figure 6:**
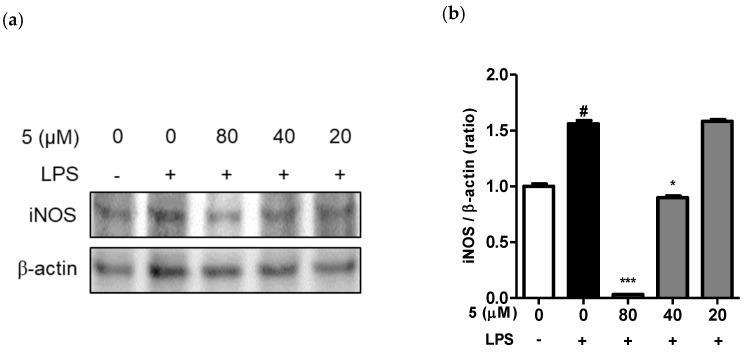
Effect of 3-(3-hydroxyphenyl)-indolin-2-one on iNOS protein expression in lipopolysaccharide (LPS)-stimulated RAW264.7 cells; (-): non-treated group, (+): LPS-treated group. (**a**) RAW264.7 macrophages (2.0 × 10^6^ cells/6-cm dish) were treated with the indicated concentrations of 3-(3-hydroxyphenyl)-indolin-2-one (20, 40, and 80 µM) for 2 h and then stimulated with LPS (500 ng/mL) for 12 h. The control group was treated with dimethyl sulfoxide at 0.05%. iNOS protein level was measured by immunoblotting with the specific iNOS antibody. (**b**) Bar chart of the intensity of immunoblot bands visualized using Image J software. Data are presented as the mean ± standard deviation (SD) of three independent experiments. White bar: non-treated group, black bar: LPS treated group, gray bar: sample treated group. ^#^ *p* < 0.0001 vs. the control group. *** *p* < 0.0001 or * *p* < 0.05 vs. the LPS group.

Therefore, we attempted to confirm the inhibition of LPS-induced iNOS protein expression by 3-(3-hydroxyphenyl)-indolin-2-one. RAW264.7 cells were treated with various concentrations of 3-(3-hydroxyphenyl)-indolin-2-one for 2 h and then treated with LPS for 12 h. As shown in Figure 6a, iNOS protein expression was strongly suppressed by 3-(3-hydroxyphenyl)-indolin-2-one treatment in a concentration-dependent manner, similar to that observed for nitric oxide and iNOS mRNA levels. The bar chart displays the intensity of the immunoblot bands visualized using Image J software. In addition, this result correlated with the inhibition of nitric oxide (Figure 4b) and mRNA expression of iNOS (Figure 5b). 

### 2.4. Effect of 3-(3-Hydroxyphenyl)-Indolin-2-One on Protein Kinase B (Akt), MAPKs, and NF-κB Phosphorylation in LPS-Induced RAW264.7 Cells

The Akt, mitogen-activated protein kinases (MAPKs), and NF-κB pathways are representative signaling pathways that mediate cell proliferation, differentiation, and apoptosis in many cells. As external stimuli, such as LPS, mediate the immune response, intracellular signaling pathways activate and promote the secretion of TNF-α, IL-6, and nitric oxide in immune cells [3,5,7,9].

To elucidate the molecular mechanisms underlying the anti-inflammatory signaling pathway induced by 3-(3-hydroxyphenyl)-indolin-2-one, we first investigated the phosphorylation of Akt and MAPK (ERK, JNK, p38) proteins, which are representative signaling proteins activated by LPS in RAW264.7 cells.

**Figure 7:**
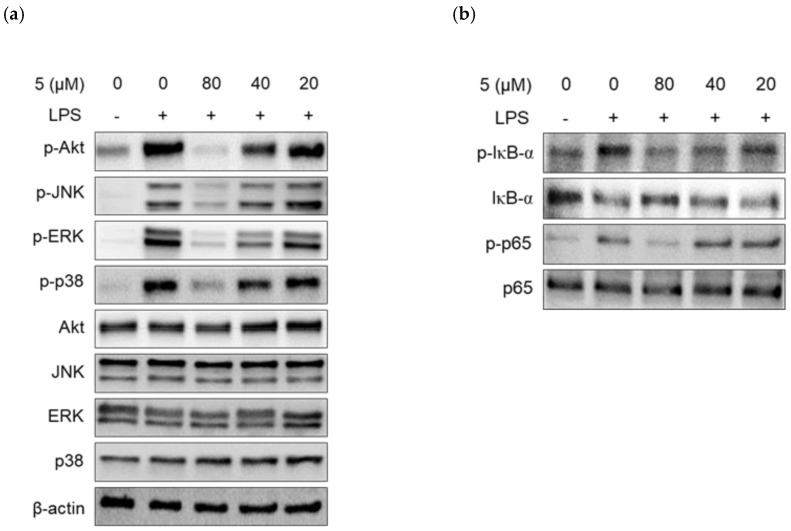

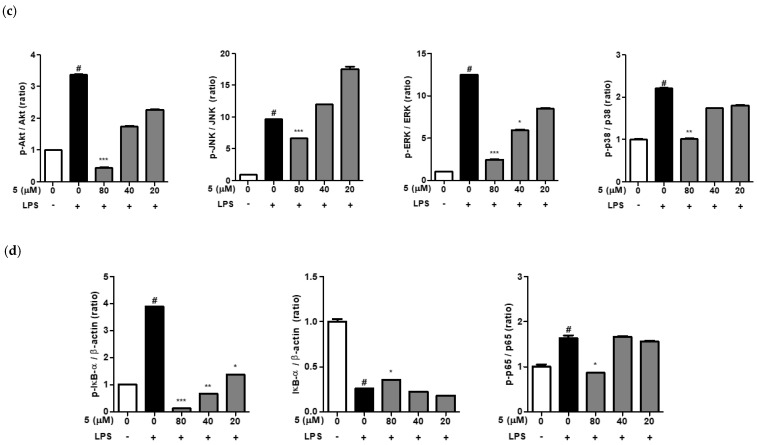
Effects of 3-(3-hydroxyphenyl)-indolin-2-one on phosphorylation of Akt, MAPKs proteins, and NF-κB pathways in RAW264.7 cells; (-): non-treated group, (+): LPS-treated group. (**a**,**b**) RAW264.7 cells (2.0 × 10^6^ cells/6-cm dish) treated with the indicated concentrations of 3-(3-hydroxyphenyl)-indolin-2-one (20–80 µg/mL) for 2 h and then stimulated with lipopolysaccharide (LPS; 500 ng/mL) for 30 min. Whole-cell lysates were immunoblotted with the specific antibodies indicated on the left side of each panel. The level of β-actin was measured as an internal loading control. (**c**) pAKT, pJNK, pERK, and pp38 protein levels were validated and total protein levels were obtained using the ImageJ software. (**d**) pIκB, IκB, and p65 protein levels were validated using the Image J software. β-actin was used as an internal control. Data are presented as the mean ± standard deviation (SD) of three independent experiments. White bar: non-treated group, black bar: LPS treated group, gray bar: sample treated group. ^#^ *p* < 0.0001 vs. the control group. *** *p* < 0.0001, ** *p* < 0.001 or * *p* < 0.05 vs. the LPS group.

As shown in Figure 7a, phosphorylation of MAPK (JNK, ERK, p38) and Akt was induced after 30 min of LPS treatment and was strongly inhibited by 3-(3-hydroxyphenyl)-indolin-2-one treatment at 80 μM. We also confirmed that 3-(3-hydroxyphenyl)-indolin-2-one inhibited LPS-induced phosphorylation of JNK, ERK, and p38 in a concentration-dependent manner (20–80 µM). In addition, LPS-induced phosphorylation of Akt protein was significantly inhibited in a concentration-dependent manner by 3-(3-hydroxyphenyl)-indolin-2-one treatment (Figure 7a). Next, we analyzed the NF-κB pathway, a representative pathway that induces LPS-induced inflammation. When a cell is in a normal state, IκBα protein exists in a complex with p65 and p50 proteins in the cytoplasm; however, when stimulated by LPS, IκBα is phosphorylated and then degraded [7]. At the same time, p65 is phosphorylated and translocated into the nucleus to act as a transcription factor. As shown in Figure 7b, when cells were treated with LPS, IκBα phosphorylation, IκBα degradation, and p65 phosphorylation were observed. However, treatment with 3-(3-hydroxyphenyl)-indolin-2-one strongly suppressed IκBα phosphorylation, IκBα degradation, and p65 phosphorylation in a concentration-dependent manner in RAW264.7 cells (Figure 7b). Taken together, these results indicate that 3-(3-hydroxyphenyl)-indolin-2-one inhibits the production of inflammatory factors such as nitric oxide, IL-6, and TNF-α by strongly inhibiting the phosphorylation of Akt, MAPKs (JNK, ERK, and p38), and the NF-κB pathway (Figure 7).

## 3. Discussion

Inflammation is a defense mechanism against harmful stimuli in the body. When cells and tissues are stimulated externally, vasoactive substances are released locally, causing inflammation, fever, pain, and loss of function [39]. NSAIDs with analgesic, antipyretic, and anti-inflammatory actions are commonly used globally in acute and chronic inflammatory conditions [40]. NSAIDs are commonly used to treat various types of diseases related to inflammation, pain, and fever by reducing the synthesis of prostaglandins via blocking the COX enzyme [41]. However, their extensive use has associated side effects such as gastrointestinal disorders and edema [42]. Accordingly, many studies have been conducted in recent years to discover new active materials with low toxicity and excellent anti-inflammatory action using natural product derivatives [43,44].

In this study, nineteen 3-substituted-indolin-2-one derivatives were synthesized to identify anti-inflammatory materials. In addition, 3-(3-hydroxyphenyl)-indolin-2-one, which had the highest activity among the 19 derivatives, was selected owing to its inhibition of nitric oxide production. To investigate the anti-inflammatory mechanism of 3-(3-hydroxyphenyl)-indolin-2-one, IL-6 and TNF-α cytokine production and mRNA expression were analyzed, as were the Akt, MAPKs, and NF-κB signaling pathways.

The nineteen 3-subsituted-indolin-2-one derivatives were examined at various concentrations (10–80 µM) based on their ability to preserve cell viability and induce the inhibitory activity of nitric oxide using RAW264.7 cells. Among the nineteen 3-subsituted-indolin-2-one derivatives, 3-(3-hydroxyphenyl)-indolin-2-one showed the lowest cytotoxicity and was confirmed to exhibit excellent NO inhibitory activity. Activated macrophages produce inflammatory mediators such as NO, TNF-α, and IL-6, and increase the inflammatory response at the early stage of infection [45]. TNF-α is a representative cytokine plays an important role in the inflammatory response, and induces the production of other cytokines to sustain the inflammatory response [46,47]. Fever symptoms appear when IL-6 is excessively produced in an inflammatory response to LPS. The production of TNF-α and IL-6 in LPS-stimulated RAW264.7 cells was decreased in a concentration-dependent manner compared with the LPS-treated group using 3-(3-hydroxyphenyl)-indolin-2-one treatment at 20, 40, and 80 µM. This result is consistent with previous studies, and the inflammatory response in the early stages of infection can be effectively controlled by regulating the production of TNF-α and IL-6. These results imply that the regulation of MAPKs and NF-kB suppresses the inflammatory response [48,49]. In macrophages, iNOS, overexpressed by LPS, produces nitric oxide, intensifying the inflammatory response. Analysis of iNOS expression in LPS-stimulated RAW264.7 cells showed that iNOS expression was decreased in a concentration-dependent manner by 3-(3-hydroxyphenyl)-indolin-2-one treatment at 40 or 80 µM. Therefore, 3-(3-hydroxyphenyl)-indolin-2-one is expected to ultimately reduce NO production by inhibiting iNOS expression in RAW264.7 cells. The MAPK pathway is activated by external stimuli, and various intracellular responses occur through the phosphorylation of specific substrates in the cell. ERK, JNK, and p38 belong to the MAPK subfamily. p38 and SAPK are protein kinases belonging to the MAP kinase family [50,51,52]. SAPK is also called JNK because it can phosphorylate the N-terminal region of c-Jun. p38 and JNK kinases are activated by inflammatory cytokines, such as TNF-α and IL-1β, various stress stimuli, ultraviolet rays, and chromosome-damaging substances, and induce various biological responses, such as cell differentiation, apoptosis, and inflammatory responses. ERK, another MAP kinase, is involved in the signaling pathways that promote cell growth and differentiation [50,51,52]. Analysis of MAPK phosphorylation and the NF-κB pathway by LPS shows that the increased phosphorylation of MAPKs, NF-κB subunit p65, and IκBα, along with degradation of IκBα are significantly prevented by treatment with 3-(3-hydroxyphenyl)-indolin-2-one in a concentration-dependent manner in RAW264.7 cells. During the inflammatory response, NF-κB regulates the expression of iNOS and COX-2. p65 protein acts as a transcriptional regulator and increases the expression of iNOS and COX-2 to regulate the inflammatory response [53,54]. Therefore, we hypothesize that the inhibition of phosphorylation and degradation of IκBα and phosphorylation of p65 induced by 3-(3-hydroxyphenyl)-indolin-2-one decrease the expression of iNOS.

## 4. Conclusions

Considering the above results, 3-(3-hydroxyphenyl)-indolin-2-one was confirmed as a non-toxic compound in the RAW264.7 cell line. Moreover, it was verified that 3-(3-hydroxyphenyl)-indolin-2-one effectively inhibits pro-inflammatory mediator (NO, TNF-α, IL-6) production by LPS stimulation. In addition, the intracellular signal transduction of anti-inflammatory activity of 3-(3-hydroxyphenyl)-indolin-2-one was attenuated by regulating the Akt, MAPK, and NF-κB signaling pathways in RAW264.7 cells. These results show that 3-(3-hydroxyphenyl)-indolin-2-one could be developed as a drug for the prevention of diseases based on inflammation (e.g., arthritis, inflammatory bowel disease, inflammatory skin disease, gastritis, etc.). However, these experiments were performed by cell lines in vitro; further research applied to animal experiments will be conducted to verify the results.

## 5. Materials and Methods

### 5.1. Antibodies and Reagents

Phosphorylation-specific antibodies against JNK, ERK1/2, p38, IκBα, and p65, and antibodies against JNK, ERK1/2, p38, IκBα, p65, iNOS, glyceraldehyde 3-phosphate dehydrogenase, and β-actin were purchased from Cell Signaling Technology (Danvers, MA, USA). Mouse interleukin-6 (IL-6) and TNF-α enzyme-linked immunosorbent assay (ELISA) kits were obtained from BD Biosciences (San Jose, CA, USA). EZ-Cytox reagent was purchased from DoGen Bio (Seoul, Republic of Korea). Sulfanilamide, phosphoric acid, and naphthyl ethylenediamine dihydrochloride were obtained from Sigma-Aldrich (St. Louis, MO, USA). Lipopolysaccharides from *E. coli* 0111:B4 (LPS) were obtained from Sigma-Aldrich (St. Louis, MO, USA). 

### 5.2. Cell Culture

RAW264.7 cells (Korean Cell Line Bank, Seoul, Republic of Korea) were cultured in Dulbecco’s modified Eagle’s medium (Corning, NY, USA) supplemented with 10% fetal bovine saline and 1% penicillin and streptomycin. Cell growth conditions were determined at 37 °C and humidified at 5% CO_2_.

### 5.3. Cell Viability

RAW264.7 macrophage cells were seeded into 96-well plates. The nineteen 3-substituted-indolin-2-one derivatives were diluted with culture medium to 20, 40, and 80 μM concentrations and then treated with cells for 2 h. Subsequently, the cells were treated with LPS (500 ng/mL) for 20 h. Cell viability was determined using the EZ-Cytox reagent. Absorbance was measured at 450 nm using a microplate reader.

### 5.4. Determination of Nitric Oxide (NO) 

NO production from cell culture supernatants treated with nineteen 3-substituted-indolin-2-one derivatives was analyzed using the Griess method. Briefly, cells were stimulated with 20, 40, and 80 μM of the nineteen 3-substituted-indolin-2-one derivatives for 2 h and then treated with LPS (500 ng/mL) for 20 h. Nitric oxide in the cell supernatants was reacted with an equal volume of Griess reagent.

### 5.5. Determination of Cytokines TNF-α and IL-6

To determine the secretion of TNF-α and IL-6, RAW264.7 cells were seeded into 96-well plates. The next day, cells were treated with different concentrations (20, 40, and 80 μM) of 3-(3-hydroxyphenyl)-indolin-2-one for 2 h, followed by pretreatment with 500 ng/mL LPS for 20 h. Cell supernatants were collected, and cytokines (TNF-α and IL-6) were analyzed using the respective ELISA sets (R&D systems, MN, USA). 

### 5.6. Preparation of Cell Lysate and Immunoblotting

RAW264.7 cells were plated into a 6-well plate (2 × 10^6^ cells/well) and then treated with 3-(3-hydroxyphenyl)-indolin-2-one for 2 h, followed by treatment with LPS for 30 min (for NF-κB p65, IκB-α and MAPKs proteins) or 18 h (for iNOS protein). Then, washed cells were lysed using radioimmunoprecipitation assay buffer containing a phosphatase inhibitor cocktail (Sigma-Aldrich, St. Louis, MO, USA), dithiothreitol (Wako, Tokyo, Japan), and complete™ Mini Protease Inhibitor Cocktail (Roche Diagnostics Corp., IN, USA). The soluble proteins from cell lysates were collected after centrifugation at 13,000 rpm for 20 min at 4 °C. The SDS-PAGE separated the proteins, and proteins were transferred to a polyvinylidene fluoride (PVDF) membrane and blocked using skim milk at 4 °C overnight. The PVDF membranes were incubated with specific primary antibodies, diluted with TBS with Tween-20 (0.5%). The membranes were washed three times with TBS-T buffer, followed by treatment with a secondary antibody linked to horseradish peroxidase (HRP). Protein was detected using Super Signal^®^ West Femto Substrate (Thermo Fisher, CA, USA) and developed using a Fusion Solo Chemiluminescence System (Vilber Lourmat, Paris, France) ECL detection system.

### 5.7. Quantitative Real-Time Reverse Transcription Polymerase Chain Reaction (qRT-PCR) 

RAW264.7 cells were plated into a 6-well plate (2 × 10^6^ cells/well) and then treated with 3-(3-hydroxyphenyl)-indolin-2-one for 2 h, followed by treatment with LPS (500 ng/mL) for 6 h (for IL-6, TNF-α, and iNOS mRNA expression). Total RNA was isolated using the RNeasy Mini Kit (Qiagen, CA, USA), and cDNA was synthesized using the AccuPower revese transcriptase Premix Kit (Bioneer, Daejon, Republic of Korea). qRT-PCR quantification was analyzed using TaqMan Fast Advanced Master mix with iNOS (Mm00440502_m1), IL-6 (Mm00446190_m1), TNF-α (Mm00443258_m1) and GAPDH (Mm99999915_g1) TaqMan primer sets (Applied Biosystems, CA, USA). Amplification conditions were determined by the Quant 3 PCR system (Applied Biosystems).

### 5.8. Statistical Analysis

The results are expressed as mean ± standard deviation of triplicate experiments. Results were analyzed statistically using the Mann–Whitney U-test in GraphPad Prism 8 (GraphPad Software, San Diego, CA, USA), with *p* < 0.05 considered statistically significant.

## Supplementary Materials

The following supporting information can be downloaded at: https://www.mdpi.com/article/10.3390/ijms24032066/s1.
