# Supplementary material for: Design, Synthesis, and Biological Evaluation of 3-Substituted-Indolin-2-One Derivatives as Potent Anti-Inflammatory Agents
Source: Int J Mol Sci. 2023 Jan 20;24(3):2066. doi: 10.3390/ijms24032066 (PMC9916847; doi:10.3390/ijms24032066)
Supplement: Supplementary file 1 [file ijms-24-02066-s001.zip › ijms-2138405-supplementary.pdf]

Supplementary Materials

Figure S1 : Supporting information

Design, Synthesis, and Biological Evaluation of Isoaurone Derivatives as Potent Anti-inflammatory Agents

Sung Jin Kim[1,#], Sang Heck Lee[2,#], Heesu Lee[3], Myoung-Sook Shin[1,*] and Jae Wook Lee[2,*]

[1] College of Korean Medicine, Gachon University, Seongnam-si, Gyeonggi-do 13120, Korea
[2] Natural Product Research Center, Korea Institute of Science and Technology (KIST), Gangneung 25451, Republic of Korea
[3] Department of Anatomy, College of Dentistry, Gangneung Wonju National University (GWNU), Gangneung 25457, Republic of Korea

[#]These authors equally contributed in this work
* Correspondence: ms.shin@gachon.ac.kr; Tel.: +82-31-750-5423 (M.-S. S); jwlee5@kist.re.kr; Tel.: +82-33-650-3514 (J.W.L)

Experimental Section

All solvent and starting materials, including anhydrous solvents and chemicals, were purchased from commercial vendors and used without further purification.

Commercially available chemicals and solvents were used as received without further purification, unless specified. Organic solutions were concentrated under reduced pressure on rotary evaporator using a water bath. Reaction was heated by IKA® RCT basic stir plate. Reaction was monitored by TLC (silica gel 60 F_{254}, Sigma Aldrich) and LC/MS (Shimadzu, LCMS-2020). Nuclear magnetic resonance (^{1}H NMR, ^{13}C NMR) spectra were recorded on a Varian 500MHz Superconducting LC-FT-NMR spectrometer. Chemical shift (δ values) and coupling constants (*J* values) are given in ppm and Hz, respectively, using tetramethylsilane as an internal standard. Preparative reverse-phase HPLC was carried out on C18 column (YMC-Pack Pro C18, 250 x 20 mml. D, S-10 µm, 12 nm) with ACN/water gradients containing 0.05% formic acid. The final structures were fully characterized by 1H NMR, 13C NMR, and LC-MS. The purities of the compound were analyzed by LC-MS (Shimadzu, LCMS-2020), and all final compounds exhibited the purity greater than 95% for biological evaluation. The detail synthetic procedure and the spectra of compounds are included in the supporting information.

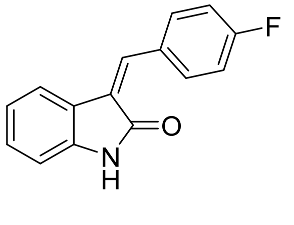

(Z)-3-(4-fluorobenzylidene) indolin-2-one

Indolin-2-one (39.0 mg, 0.3 mmol) was dissolved in methanol (1 mL). 4-fluorobenzaldehyde (52.2 mg, 0.3 mmol) and piperidine (30.0 µL, 0.36 mmol) was added to reaction mixture sequentially. The reaction mixture was refluxed at 80 °C for 8 h. After completion of the reaction, the reaction mixture was cooled to room temperature (15-25 °C), and then the reaction mixture was concentrated under reduced pressure. The residue was dissolved in

ethyl acetate (50 mL), and H_2O (30 mL). The solution was transferred to separatory funnel, organic layers were separated. The organic layer was dried over Na_2SO_4, and filtered. The solvent was removed under reduced pressure and residue was purified by prep-LC (ACN / H_2O, 10% ACN to 100% ACN for 1 h, linear gradient) to give the yellow solid (22.3 mg, 0.09 mmol, 30.1%). ^{1}H NMR (500 MHz, $CDCl_3$) δ 9.05 (s, 1H), 8.33 (t, J = 5.8 Hz, 1H), 7.78 (s, 1H), 7.66 (t, J = 5.4 Hz, 2H), 7.59 (d, J = 7.7 Hz, 1H), 7.50 (t, J = 4.35 Hz, 1H), 7.22 (td, J = 7.8, 1.3 Hz, 1H), 7.16 (t, J = 8.75 Hz, 2H), 6.92 (d, J = 7.8 Hz, 1H), 6.89 (t, J = 7.65 Hz, 1H). ^{13}C NMR (125 MHz, $CDCl_3$) δ 170.5, 162.4, 141.9, 139.9, 136.4, 131.6, 131.5, 130.2, 123.0, 122.0, 116.1, 115.9, 110.6. MS (m/z): $[M + H]^+$ calcd for $C_{15}H_{10}FNO$, 240.07; found 240.1.

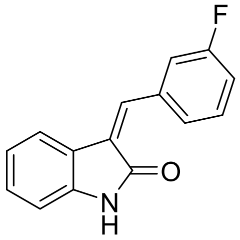

(Z)-3-(3-fluorobenzylidene) indolin-2-one

Indolin-2-one (39.0 mg, 0.3 mmol) was dissolved in methanol (1 mL). 3-fluorobenzaldehyde (52.2 mg, 0.3 mmol) and piperidine (30.0 μL, 0.36 mmol) was added to reaction mixture sequentially. The reaction mixture was refluxed at 80 °C and stirred for 8 h. After completion of the reaction, the reaction mixture was cooled to room temperature (15-25 °C), and then the reaction mixture was concentrated under reduced pressure. The residue was dissolved in EA (50 mL), and H_2O (30 mL). The solution was transferred to separatory funnel, organic layers were separated. The organic layer was dried over Na_2SO_4, and filtered. The solvent was removed under reduced pressure and residue was purified by prep-LC (ACN / H_2O, 10% ACN to 100% ACN for 1 h, linear gradient) to give the yellow solid (26.8 mg, 0.11 mmol, 36.7%). ^{1}H NMR (500 MHz, $CDCl_3$) δ 8.23 (s, 1H), 7.75 (s, 1H), 7.58 (d, J = 7.55 Hz, 1H), 7.45-7.43 (m, 2H), 7.36-7.33 (m, 1H), 7.23 (td, J = 7.7, 1.15 Hz, 1H), 7.15-7.11 (m, 1H), 6.89 (t, J = 7.75 Hz, 2H). ^{13}C NMR (125 MHz, $CDCl_3$) δ 169.8, 163.9, 161.9, 141.7, 137.1, 135.8, 130.4, 128.5, 125.1, 123.3, 122.2, 121.5, 116.8, 116.2, 110.4. MS (m/z): $[M + H]^+$ calcd for $C_{15}H_{10}FNO$, 240.08; found 240.1.

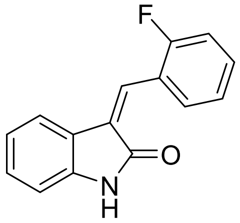

(Z)-3-(2-fluorobenzylidene) indolin-2-one

Indolin-2-one (39.0 mg, 0.3 mmol) was dissolved in methanol (1 mL). 2-fluorobenzaldehyde (52.2 mg, 0.3 mmol) and piperidine (30.0 μL, 0.36 mmol) was added to reaction mixture sequentially. The reaction mixture was refluxed at 80 °C and stirred for 8 h. After completion of the reaction, the reaction mixture was cooled to room temperature (15-25 °C), and then the reaction mixture was concentrated under reduced pressure. The residue was dissolved in EA (50 mL), and H_2O (30 mL). The solution was transferred to separatory funnel, organic layers were separated. The organic layer was dried over Na_2SO_4, and filtered. The solvent was removed under reduced pressure and residue was purified by prep-LC (ACN / H_2O, 10% ACN to 100% ACN for 1 h, linear gradient) to give the yellow solid (21.5 mg, 0.08 mmol, 26.7%). ^{1}H NMR (500 MHz, $CDCl_3$) δ 8.17 (s, 1H), 7.81 (s, 1H), 7.71 (t, J = 7.5 Hz, 1H), 7.44 (d, J = 7.5 Hz, 2H), 7.24-7.17 (m, 3H), 6.89-6.84 (m, 2H). ^{13}C NMR (125 MHz, $CDCl_3$) δ 169.5, 161.7, 141.8, 131.8, 130.5, 130.4, 129.9, 124.2, 123.5, 122.1, 121.7, 120.1, 116.4, 110.3, 109.7. MS (m/z): $[M + H]^+$ calcd for $C_{15}H_{10}FNO$, 240.08; found 240.1.

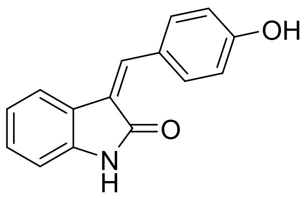

(Z)-3-(4-hydroxybenzylidene)indolin-2-one

Indolin-2-one (39.0 mg, 0.3 mmol) was dissolved in methanol (1 mL). 4-hydroxybenzaldehyde (36.6 mg, 0.3 mmol) and piperidine (30.0 µL, 0.36 mmol) was added to reaction mixture sequentially. The reaction mixture was refluxed at 80 °C and stirred for 8 h. After completion of the reaction, the reaction mixture was cooled to room temperature (15-25 °C), and then the reaction mixture was concentrated under reduced pressure. The residue was dissolved in EA (50 mL), and H_2O (30 mL). The solution was transferred to separatory funnel, organic layers were separated. The organic layer was dried over Na_2SO_4, and filtered. The solvent was removed under reduced pressure and residue was purified by prep-LC (ACN / H_2O, 10% ACN to 100% ACN for 1 h, linear gradient) to give the yellow solid (24.6 mg, 0.10 mmol, 33.3%). ^{1}H NMR (500 MHz, DMSO-d_6) δ 10.55 (s, 1H), 7.68 (s, 1H), 7.60 (dd, *J* = 7.75, 1.75 Hz, 1H), 7.48 (d, *J* = 7.15 Hz, 1H), 7.30 (td, *J* = 6.75, 1.75 Hz, 1H), 7.20 (td, *J* = 7.75, 1.15 Hz, 1H), 6.96 (d, *J* = 8.25, 1H), 6.91 (td, *J* = 7.5, 1.1 Hz, 1H), 6.87 (d, *J* = 8 Hz, 1H), 6.84 (td, *J* = 7.65, 1.1 Hz, 1H). ^{13}C NMR (125 MHz, DMSO-d_6) δ 169.1, 156.7, 142.7, 132.8, 131.9, 129.9, 129.8, 126.7, 122.5, 121.5, 121.5, 121.3, 119.1, 116.2, 110.2. MS (m/z): $[M + H]^+$ calcd for $C_{15}H_{11}NO_2$, 238.08; found 238.1.

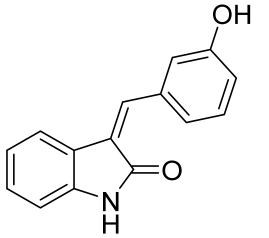

(Z)-3-(3-hydroxybenzylidene)indolin-2-one

Indolin-2-one (39.0 mg, 0.3 mmol) was dissolved in methanol (1 mL). 3-hydroxybenzaldehyde (36.6 mg, 0.3 mmol) and piperidine (30.0 µL, 0.36 mmol) was added to reaction mixture sequentially. The reaction mixture was refluxed at 80 °C and stirred for 8 h. After completion of the reaction, the reaction mixture was cooled to room temperature (15-25 °C), and then the reaction mixture was concentrated under reduced pressure. The residue was dissolved in EA (50 mL), and H_2O (30 mL). The solution was transferred to separatory funnel, organic layers were separated. The organic layer was dried over Na_2SO_4, and filtered. The solvent was removed under reduced pressure and residue was purified by prep-LC (ACN / H_2O, 10% ACN to 100% ACN for 1 h, linear gradient) to give the yellow solid (23.4 mg, 0.10 mmol, 33.3%). ^{1}H NMR (500 MHz, DMSO-d_6) δ 10.60 (s, 1H), 7.68 (s, 1H), 7.57 (d, *J* = 7.65 Hz, 1H), 7.53 (s, 1H), 7.31 (t, *J* = 7.85 Hz, 1H), 7.24-7.20 (m, 2H), 7.08 (t, *J* = 8.3 Hz, 2H), 6.88-6.84 (m, 2H). ^{13}C NMR (125 MHz, DMSO-d_6) δ 169.0, 157.6, 143.0, 136.3, 135.8, 130.4, 130.2, 127.6, 122.8, 121.4, 121.1, 120.4, 117.1, 115.7, 110.4. MS (m/z): $[M + H]^+$ calcd for $C_{15}H_{11}NO_2$, 238.08; found 238.1.

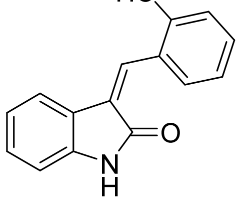

(Z)-3-(2-hydroxybenzylidene)indolin-2-one

Indolin-2-one (39.0 mg, 0.3 mmol) was dissolved in methanol (1 mL). 2-hydroxybenzaldehyde (36.6 mg, 0.3 mmol) and piperidine (30.0 μL, 0.36 mmol) was added to reaction mixture sequentially. The reaction mixture was refluxed at 80 °C and stirred for 8 h. After completion of the reaction, the reaction mixture was cooled to room temperature (15-25 °C), and then the reaction mixture was concentrated under reduced pressure. The residue was dissolved in EA (50 mL), and H_2O (30 mL). The solution was transferred to separatory funnel, organic layers were separated. The organic layer was dried over Na_2SO_4, and filtered. The solvent was removed under reduced pressure and residue was purified by prep-LC (ACN / H_2O, 10% ACN to 100% ACN for 1 h, linear gradient) to give the yellow solid (26.6 mg, 0.11 mmol, 36.7%). ^1H NMR (500 MHz, DMSO-d_6) δ 10.52 (s, 1H), 8.37 (d, J = 8.95 Hz, 1H), 7.68 (d, J = 8.15 Hz, 1H), 7.60 (d, J = 7.65 Hz, 2H), 7.53 (s, 1H), 7.20 (td, J = 7.65, 1.1 Hz, 1H), 6.90-6.86 (m, 3H), 6.81 (t, J = 7.6 Hz, 1H). ^{13}C NMR (125 MHz, DMSO-d_6) δ 169.4, 159.6, 142.6, 136.9, 135.0, 132.1, 129.7, 125.1, 124.8, 122.2, 121.5, 121.3, 115.9, 115.4, 110.2. MS (m/z): $[M + H]^+$ calcd for $C_{15}H_{11}NO_2$, 238.08; found 238.1.

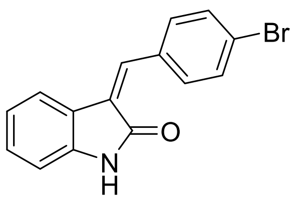

(Z)-3-(4-bromobenzylidene) indolin-2-one

Indolin-2-one (39.0 mg, 0.3 mmol) was dissolved in methanol (1 mL). 4-bromobenzaldehyde (55.2 mg, 0.3 mmol) and piperidine (30.0 μL, 0.36 mmol) was added to reaction mixture sequentially. The reaction mixture was refluxed at 80 °C and stirred for 8 h. After completion of the reaction, the reaction mixture was cooled to room temperature (15-25 °C), and then the reaction mixture was concentrated under reduced pressure. The residue was dissolved in EA (50 mL), and H_2O (30 mL). The solution was transferred to separatory funnel, organic layers were separated. The organic layer was dried over Na_2SO_4, and filtered. The solvent was removed under reduced pressure and residue was purified by prep-LC (ACN / H_2O, 10% ACN to 100% ACN for 1 h, linear gradient) to give the yellow solid (25.7 mg, 0.086 mmol, 28.7%). ^1H NMR (500 MHz, $CDCl_3$) δ 8.38 (s, 1H), 8.15 (d, J = 8.35 Hz, 1H), 7.72 (s, 1H), 7.61 (d, J = 6.1 Hz, 2H), 7.57 (d, J = 7.95 Hz, 2H), 7.53 (d, J = 8.3 Hz, 2H), 7.23 (t, J = 7.8 Hz, 1H), 6.90-6.88 (m, 2H). ^{13}C NMR (125 MHz, $CDCl_3$) δ 170.0, 141.8, 136.0, 133.6, 132.1, 131.7, 131.0, 130.4, 128.2, 123.2, 122.1, 119.6, 110.4. MS (m/z): $[M + H]^+$ calcd for $C_{15}H_{10}BrNO$, 299.99; found 300.0.

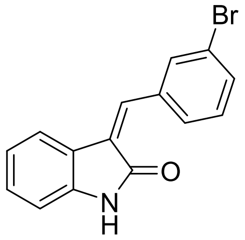

(Z)-3-(3-bromobenzylidene) indolin-2-one

Indolin-2-one (39.0 mg, 0.3 mmol) was dissolved in methanol (1 mL). 3-bromobenzaldehyde (55.2 mg, 0.3 mmol) and piperidine (30.0 μL, 0.36 mmol) was added to reaction mixture sequentially. The reaction mixture was refluxed at 80 °C and stirred for 8 h. After completion of the reaction, the reaction mixture was cooled to room temperature (15-25 °C), and then the reaction mixture was concentrated under reduced pressure. The residue was dissolved in EA (50 mL), and H_2O (30 mL). The solution was transferred to separatory funnel, organic layers were separated. The organic layer was dried over Na_2SO_4, and filtered. The solvent was removed under reduced pressure and residue was purified by prep-LC (ACN / H_2O, 10% ACN to 100% ACN for 1 h, linear gradient) to give the yellow solid (20.7 mg, 0.069 mmol, 23.7%). ^1H NMR (500 MHz, $CDCl_3$) δ 8.86 (s, 1H), 7.68 (s, 1H), 7.62 (s, 1H), 7.47-7.42 (m, 3H), 7.24 (t, J = 7.9 Hz, 1H), 7.13 (t, J = 7.7 Hz, 1H), 6.83-6.77 (m, 2H). ^{13}C NMR (125 MHz, $CDCl_3$) δ 169.9, 141.9, 137.1, 135.5, 132.6, 132.0, 130.5, 130.4, 128.8, 127.9, 123.3, 122.9, 122.2, 121.5,

110.5. MS (m/z): $[M + H]^+$ calcd for $C_{15}H_{10}BrNO$, 299.99; found 300.0.

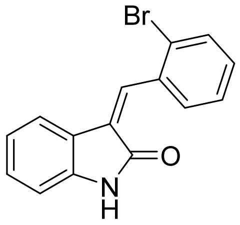

(Z)-3-(2-bromobenzylidene) indolin-2-one

Indolin-2-one (39.0 mg, 0.3 mmol) was dissolved in methanol (1 mL). 2-bromobenzaldehyde (55.2 mg, 0.3 mmol) and piperidine (30.0 µL, 0.36 mmol) was added to reaction mixture sequentially. The reaction mixture was refluxed at 80 °C and stirred for 8 h. After completion of the reaction, the reaction mixture was cooled to room temperature (15-25 °C), and then the reaction mixture was concentrated under reduced pressure. The residue was dissolved in EA (50 mL), and H_2O (30 mL). The solution was transferred to separatory funnel, organic layers were separated. The organic layer was dried over Na_2SO_4, and filtered. The solvent was removed under reduced pressure and residue was purified by prep-LC (ACN / H_2O, 10% ACN to 100% ACN for 1 h, linear gradient) to give the yellow solid (28.1 mg, 0.094 mmol, 31.3%). ^1H NMR (500 MHz, $CDCl_3$) δ 8.71 (s, 1H), 7.81 (s, 1H), 7.71 (d, J = 8.05 Hz, 2H), 7.40 (t, J = 7.5 Hz, 1H), 7.32-7.27 (m, 2H), 7.21 (t, J = 7.65 Hz, 1H), 6.92 (d, J = 7.75 Hz, 1H), 6.82 (t, J = 6.55 Hz, 1H). ^{13}C NMR (125 MHz, $CDCl_3$) δ 169.8, 141.9, 136.0, 135.6, 133.4, 131.0, 129.0, 127.4, 124.3, 123.4, 122.1, 121.5, 110.5. MS (m/z): $[M + H]^+$ calcd for $C_{15}H_{10}BrNO$, 299.99; found 300.0.

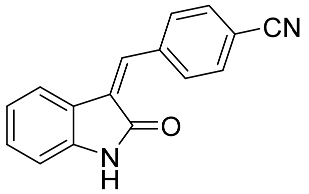

(Z)-4-((2-oxoindolin-3-ylidene) methyl) benzonitrile

Indolin-2-one (39.0 mg, 0.3 mmol) was dissolved in methanol (1 mL). 4-formylbenzonitrile (39.3 mg, 0.3 mmol) and piperidine (30.0 µL, 0.36 mmol) was added to reaction mixture sequentially. The reaction mixture was refluxed at 80 °C and stirred for 8 h. After completion of the reaction, the reaction mixture was cooled to room temperature (15-25 °C), and then the reaction mixture was concentrated under reduced pressure. The residue was dissolved in EA (50 mL), and H_2O (30 mL). The solution was transferred to separatory funnel, organic layers were separated. The organic layer was dried over Na_2SO_4, and filtered. The solvent was removed under reduced pressure and residue was purified by prep-LC (ACN / H_2O, 10% ACN to 100% ACN for 1 h, linear gradient) to give the yellow solid (23.5 mg, 0.095 mmol, 31.7%). ^1H NMR (500 MHz, $CDCl_3$) δ 8.18 (s, 1H), 7.77-7.73 (m, 5H), 7.44 (d, J = 7.7 Hz, 1H), 7.26 (t, J = 6.65 Hz, 1H), 6.89 (t, J = 7.85 Hz, 2H). ^{13}C NMR (125 MHz, $CDCl_3$) δ 169.4, 142.1, 139.8, 134.3, 132.6, 131.1, 129.9, 123.3, 122.3, 121.1, 118.5, 113.0, 110.6. MS (m/z): $[M + H]^+$ calcd for $C_{16}H_{10}N_2O$, 247.08; found 247.1.

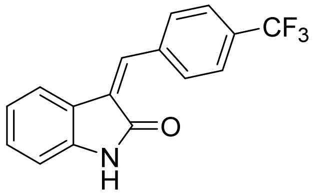

(Z)-3-(4-(trifluoromethyl) benzylidene) indolin-2-one

Indolin-2-one (39.0 mg, 0.3 mmol) was dissolved in methanol (1 mL). 4-(trifluoromethyl) benzaldehyde (52.2 mg, 0.3 mmol) and piperidine (30.0 µL, 0.36 mmol) was added to reaction mixture sequentially. The reaction

mixture was refluxed at 80 °C and stirred for 8 h. After completion of the reaction, the reaction mixture was cooled to room temperature (15-25 °C), and then the reaction mixture was concentrated under reduced pressure. The residue was dissolved in EA (50 mL), and H_2O (30 mL). The solution was transferred to separatory funnel, organic layers were separated. The organic layer was dried over Na_2SO_4, and filtered. The solvent was removed under reduced pressure and residue was purified by prep-LC (ACN / H_2O, 10% ACN to 100% ACN for 1 h, linear gradient) to give the yellow solid (24.0 mg, 0.083 mmol, 27.7%). ^{1}H NMR (500 MHz, $CDCl_3$) δ 8.86 (s, 1H), 7.80 (s, 1H), 7.75 (q, *J* = 8.45 Hz, 4H), 7.49 (d, *J* = 7.65 Hz, 1H), 7.24 (t, *J* = 5.7Hz, 1H), 6.93 (d, *J* = 7.8 Hz, 1H), 6.88 (td, *J* = 7.6, 1.05 Hz, 1H). ^{13}C NMR (125 MHz, $CDCl_3$) δ 169.7, 142.0, 138.7, 135.2, 130.7, 129.6, 129.3, 125.9, 123.3, 122.2, 121.3, 110.5 MS (m/z): $[M + H]^+$ calcd for $C_{16}H_{10}F_3NO$, 290.07; found 290.1.

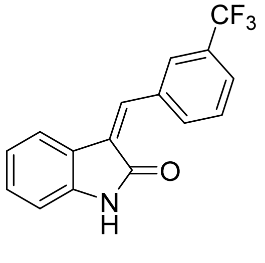

(Z)-3-(3-(trifluoromethyl) benzylidene) indolin-2-one

Indolin-2-one (39.0 mg, 0.3 mmol) was dissolved in methanol (1 mL). 3-(trifluoromethyl) benzaldehyde (52.2 mg, 0.3 mmol) and piperidine (30.0 µL, 0.36 mmol) was added to reaction mixture sequentially. The reaction mixture was refluxed at 80 °C and stirred for 8 h. After completion of the reaction, the reaction mixture was cooled to room temperature (15-25 °C), and then the reaction mixture was concentrated under reduced pressure. The residue was dissolved in EA (50 mL), and H_2O (30 mL). The solution was transferred to separatory funnel, organic layers were separated. The organic layer was dried over Na_2SO_4, and filtered. The solvent was removed under reduced pressure and residue was purified by prep-LC (ACN / H_2O, 10% ACN to 100% ACN for 1 h, linear gradient) to give the yellow solid (26.8 mg, 0.093 mmol, 31.0%). ^{1}H NMR (500 MHz, $CDCl_3$) δ 8.44 (s, 1H), 7.92 (s, 1H), 7.81 (t, *J* = 7.65 Hz, 2H), 7.69 (d, *J* = 7.85 Hz, 1H), 7.61 (t, *J* = 7.8 Hz, 1H), 7.47 (d, *J* = 7.7 Hz, 1H), 7.24 (t, *J* = 7.75 Hz, 1H), 6.92-6.86 (m, 2H). ^{13}C NMR (125 MHz, $CDCl_3$) δ 169.4, 142.1, 139.8, 134.3, 132.6, 131.1, 129.9, 123.3, 122.3, 121.1, 118.5, 113.0, 110.6. MS (m/z): $[M + H]^+$ calcd for $C_{16}H_{10}F_3NO$, 290.07; found 290.1.

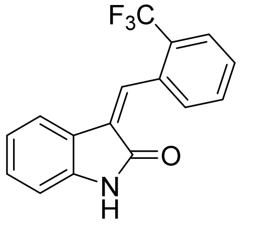

(Z)-3-(2-(trifluoromethyl) benzylidene) indolin-2-one

Indolin-2-one (39.0 mg, 0.3 mmol) was dissolved in methanol (1 mL). 2-(trifluoromethyl) benzaldehyde (52.2 mg, 0.3 mmol) and piperidine (30.0 µL, 0.36 mmol) was added to reaction mixture sequentially. The reaction mixture was refluxed at 80 °C and stirred for 8 h. After completion of the reaction, the reaction mixture was cooled to room temperature (15-25 °C), and then the reaction mixture was concentrated under reduced pressure. The residue was dissolved in EA (50 mL), and H_2O (30 mL). The solution was transferred to separatory funnel, organic layers were separated. The organic layer was dried over Na_2SO_4, and filtered. The solvent was removed under reduced pressure and residue was purified by prep-LC (ACN / H_2O, 10% ACN to 100% ACN for 1 h, linear gradient) to give the yellow solid (25.2 mg, 0.087 mmol, 29.0%). ^{1}H NMR (500 MHz, $CDCl_3$) δ 8.61 (s, 1H), 7.97 (s, 1H), 7.81 (d, *J* = 7.8 Hz, 1H), 7.71 (d, *J* = 7.55 Hz, 1H), 7.62 (t, *J* = 7.6 Hz, 1H), 7.56 (t, *J* = 8.4 Hz, 1H), 7.20 (t, *J* = 6.55 Hz, 1H), 6.96 (d, *J* = 7.7 Hz, 1H), 6.91 (d, *J* = 7.75 Hz, 1H), 6.77 (t, *J* = 7.6 Hz, 1H). ^{13}C NMR (125 MHz, $CDCl_3$) δ 169.5, 142.1, 133.4, 132.0, 130.5, 130.3, 129.3, 126.6, 123.5, 122.1, 121.4, 110.5. MS

(m/z): $[M + H]^+$ calcd for $C_{16}H_{10}F_3NO$, 290.07; found 290.1.

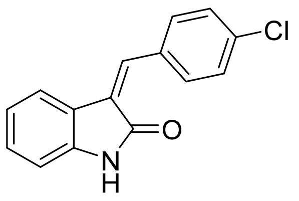

(Z)-3-(4-chlorobenzylidene) indolin-2-one

Indolin-2-one (39.0 mg, 0.3 mmol) was dissolved in methanol (1 mL). 4-chlorobenzaldehyde (42.2 mg, 0.3 mmol) and piperidine (30.0 µL, 0.36 mmol) was added to reaction mixture sequentially. The reaction mixture was refluxed at 80 °C and stirred for 8 h. After completion of the reaction, the reaction mixture was cooled to room temperature (15-25 °C), and then the reaction mixture was concentrated under reduced pressure. The residue was dissolved in EA (50 mL), and H_2O (30 mL). The solution was transferred to separatory funnel, organic layers were separated. The organic layer was dried over Na_2SO_4, and filtered. The solvent was removed under reduced pressure and residue was purified by prep-LC (ACN / H_2O, 10% ACN to 100% ACN for 1 h, linear gradient) to give the yellow solid (21.2 mg, 0.083 mmol, 27.7%). ^1H NMR (500 MHz, $CDCl_3$) δ 8.40 (s, 1H), 7.75 (s, 1H), 7.61-7.56 (m, 3H), 7.45 (d, J = 8.5 Hz, 2H), 7.23 (t, J = 7.7 Hz, 1H), 6.91-6.86 (m, 2H). ^{13}C NMR (125 MHz, $CDCl_3$) δ 170.0, 141.8, 136.0, 135.7, 133.4, 130.8, 130.3, 129.2, 128.1, 123.2, 122.1, 121.6, 110.4. MS (m/z): $[M + H]^+$ calcd for $C_{15}H_{10}ClNO$, 256.05; found 256.1.

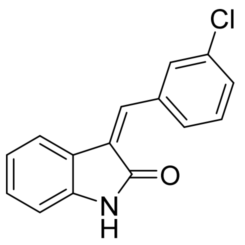

(Z)-3-(3-chlorobenzylidene) indolin-2-one

Indolin-2-one (39.0 mg, 0.3 mmol) was dissolved in methanol (1 mL). 3-chlorobenzaldehyde (42.2 mg, 0.3 mmol) and piperidine (30.0 µL, 0.36 mmol) was added to reaction mixture sequentially. The reaction mixture was refluxed at 80 °C and stirred for 8 h. After completion of the reaction, the reaction mixture was cooled to room temperature (15-25 °C), and then the reaction mixture was concentrated under reduced pressure. The residue was dissolved in EA (50 mL), and H_2O (30 mL). The solution was transferred to separatory funnel, organic layers were separated. The organic layer was dried over Na_2SO_4, and filtered. The solvent was removed under reduced pressure and residue was purified by prep-LC (ACN / H_2O, 10% ACN to 100% ACN for 1 h, linear gradient) to give the yellow solid (23.5 mg, 0.092 mmol, 30.7%). ^1H NMR (500 MHz, $CDCl_3$) δ 8.88 (s, 1H), 7.74 (s, 1H), 7.63 (s, 1H), 7.55-7.53 (m, 2H), 7.40 (d, J = 5.35 Hz, 2H), 7.23 (t, J = 7.6 Hz, 1H), 6.93 (d, J = 7.8 Hz, 1H), 6.89 (t, J = 7.6 Hz, 1H). ^{13}C NMR (125 MHz, $CDCl_3$) δ 169.9, 141.9, 136.8, 135.6, 134.9, 130.5, 130.2, 129.7, 129.2, 128.8, 127.4, 123.3, 122.2, 121.5, 110.5. MS (m/z): $[M + H]^+$ calcd for $C_{15}H_{10}ClNO$, 256.05; found 256.1.

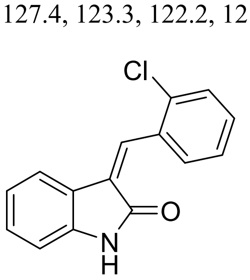

(Z)-3-(2-chlorobenzylidene) indolin-2-one

Indolin-2-one (39.0 mg, 0.3 mmol) was dissolved in methanol (1 mL). 2-chlorobenzaldehyde (42.2 mg, 0.3 mmol) and piperidine (30.0 µL, 0.36 mmol) was added to reaction mixture sequentially. The reaction mixture was refluxed at 80 °C and stirred for 8 h. After completion of the reaction, the reaction mixture was cooled to room temperature (15-25 °C), and then the reaction mixture was concentrated under reduced pressure. The residue was dissolved in EA (50 mL), and H_2O (30 mL). The solution was transferred to separatory funnel, organic layers were separated. The organic layer was dried over Na_2SO_4, and filtered. The solvent was removed under reduced pressure and residue was purified by prep-LC (ACN / H_2O, 10% ACN to 100% ACN for 1 h, linear gradient) to give the yellow solid (23.4 mg, 0.092 mmol, 30.7%). ^{1}H NMR (500 MHz, $CDCl_3$) δ 8.72 (s, 1H), 7.87 (s, 1H), 7.73 (dd, J = 7.5, 1.8 Hz, 1H), 7.52 (dd, J = 7.85, 1.4 Hz, 1H), 7.39 (td, J = 7.45, 1.8 Hz, 1H), 7.35 (dd, J = 7.5, 1.5 Hz, 1H), 7.33 (t, J = 7.85 Hz, 1H), 7.22 (td, J = 7.7, 1.2 Hz, 1H), 6.92 (d, J = 7.75 Hz, 1H), 6.83 (td, J = 7.75, 1 Hz, 1H). ^{13}C NMR (125 MHz, $CDCl_3$) δ 169.9, 142.0, 134.6, 134.0, 133.7, 130.9, 130.4, 130.3, 130.2, 129.2, 126.8. 123.4, 122.1, 121.5, 110.5. MS (m/z): $[M + H]^+$ calcd for $C_{15}H_{10}ClNO$, 256.05; found 256.1.

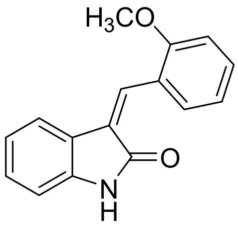

(Z)-3-(2-methoxybenzylidene)indolin-2-one

Indolin-2-one (39.0 mg, 0.3 mmol) was dissolved in methanol (1 mL). 2-methoxybenzaldehyde (42.2 mg, 0.3 mmol) and piperidine (30.0 µL, 0.36 mmol) was added to reaction mixture sequentially. The reaction mixture was refluxed at 80 °C and stirred for 8 h. After completion of the reaction, the reaction mixture was cooled to room temperature (15-25 °C), and then the reaction mixture was concentrated under reduced pressure. The residue was dissolved in EA (50 mL), and H_2O (30 mL). The solution was transferred to separatory funnel, organic layers were separated. The organic layer was dried over Na_2SO_4, and filtered. The solvent was removed under reduced pressure and residue was purified by prep-LC (ACN / H_2O, 10% ACN to 100% ACN for 1 h, linear gradient) to give the yellow solid (21.8 mg, 0.087 mmol, 29.0%). ^{1}H NMR (500 MHz, $CDCl_3$) δ 8.25 (s, 1H), 7.97 (s, 1H), 7.73 (dd, J = 7.5, 1.7 Hz, 1H), 7.56 (d, J = 7.65 Hz, 1H), 7.43 (td, J = 7.9, 1.75 Hz, 1H), 7.19 (td, J = 7.65, 1.15 Hz, 1H), 7.03 (td, J = 7.5, 1 Hz, 1H), 6.98 (dd, J = 8.25, 1 Hz, 1H), 6.88 (d, J = 7.75, 1H), 6.85 (td, J = 7.65, 1.05 Hz, 1H). ^{13}C NMR (125 MHz, $CDCl_3$) δ 170.2, 158.3, 141.4, 134.2, 131.7, 130.1, 129.7, 127.2, 123.9, 123.2, 122.2, 121.8, 120.3, 111.1, 110.1, 55.7. MS (m/z): $[M + H]^+$ calcd for $C_{16}H_{13}NO_2$, 252.09; found 252.1.

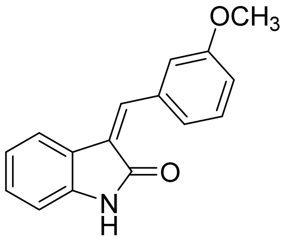

(Z)-3-(3-methoxybenzylidene)indolin-2-one

Indolin-2-one (39.0 mg, 0.3 mmol) was dissolved in methanol (1 mL). 3-methoxybenzaldehyde (42.2 mg, 0.3 mmol) and piperidine (30.0 µL, 0.36 mmol) was added to reaction mixture sequentially. The reaction mixture was refluxed at 80 °C and stirred for 8 h. After completion of the reaction, the reaction mixture was cooled to room temperature (15-25 °C), and then the reaction mixture was concentrated under reduced pressure. The residue was dissolved in EA (50 mL), and H_2O (30 mL). The solution was transferred to separatory funnel, organic layers were separated. The organic layer was dried over Na_2SO_4, and filtered. The solvent was removed under reduced pressure and residue was purified by prep-LC (ACN / H_2O, 10% ACN to 100% ACN for 1 h, linear gradient) to give the yellow solid (22.7 mg, 0.09 mmol, 30.0%). ^{1}H NMR (500 MHz, $CDCl_3$) δ 8.39 (s, 1H), 7.81 (s, 1H), 7.67 (d, J =

7.7 Hz, 1H), 7.39 (t, J = 7.85 Hz, 1H), 7.24-7.17 (m, 3H), 6.98 (dd, J = 8.25, 2.7 Hz, 1H), 6.90-6.96 (m, 2H), 3.84 (s, 3H). ^{13}C NMR (125 MHz, $CDCl_3$) δ 170.2, 159.8, 141.7, 137.6, 136.3, 130.1, 129.9, 123.5, 122.0, 121.9, 121.8, 115.8, 114.4, 110.3, 55.5. MS (m/z): $[M + H]^+$ calcd for $C_{16}H_{13}NO_2$, 252.09; found 252.1.

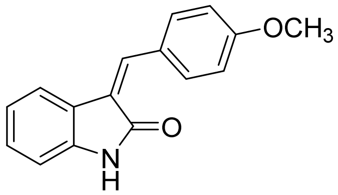

(Z)-3-(4-methoxybenzylidene)indolin-2-one

Indolin-2-one (39.0 mg, 0.3 mmol) was dissolved in methanol (1 mL). 4-methoxybenzaldehyde (42.2 mg, 0.3 mmol) and piperidine (30.0 µL, 0.36 mmol) was added to reaction mixture sequentially. The reaction mixture was refluxed at 80 °C and stirred for 8 h. After completion of the reaction, the reaction mixture was cooled to room temperature (15-25 °C), and then the reaction mixture was concentrated under reduced pressure. The residue was dissolved in EA (50 mL), and H_2O (30 mL). The solution was transferred to separatory funnel, organic layers were separated. The organic layer was dried over Na_2SO_4, and filtered. The solvent was removed under reduced pressure and residue was purified by prep-LC (ACN / H_2O, 10% ACN to 100% ACN for 1 h, linear gradient) to give the yellow solid (20.4 mg, 0.08 mmol, 26.7%). ^{1}H NMR (500 MHz, $CDCl_3$) δ 8.66 (s, 1H), 7.79 (s, 1H), 7.75 (d, J = 7.7 Hz, 1H), 7.67 (d, J = 8.6 Hz, 2H), 7.20 (td, J = 7.75, 1.2 Hz, 1H), 6.99 (d, J = 8.7 Hz, 2H), 6.92-6.88 (m, 2H), 3.89 (s, 3H). ^{13}C NMR (125 MHz, $CDCl_3$) δ 170.7, 161.1, 141.5, 137.9, 131.7, 129.6, 127.3, 125.8, 122.8, 122.2, 121.9, 114.3, 110.3, 55.6. MS (m/z): $[M + H]^+$ calcd for $C_{16}H_{13}NO_2$, 252.09; found 252.1.

1H NMR of (Z)-3-(4-fluorobenzylidene) indolin-2-one

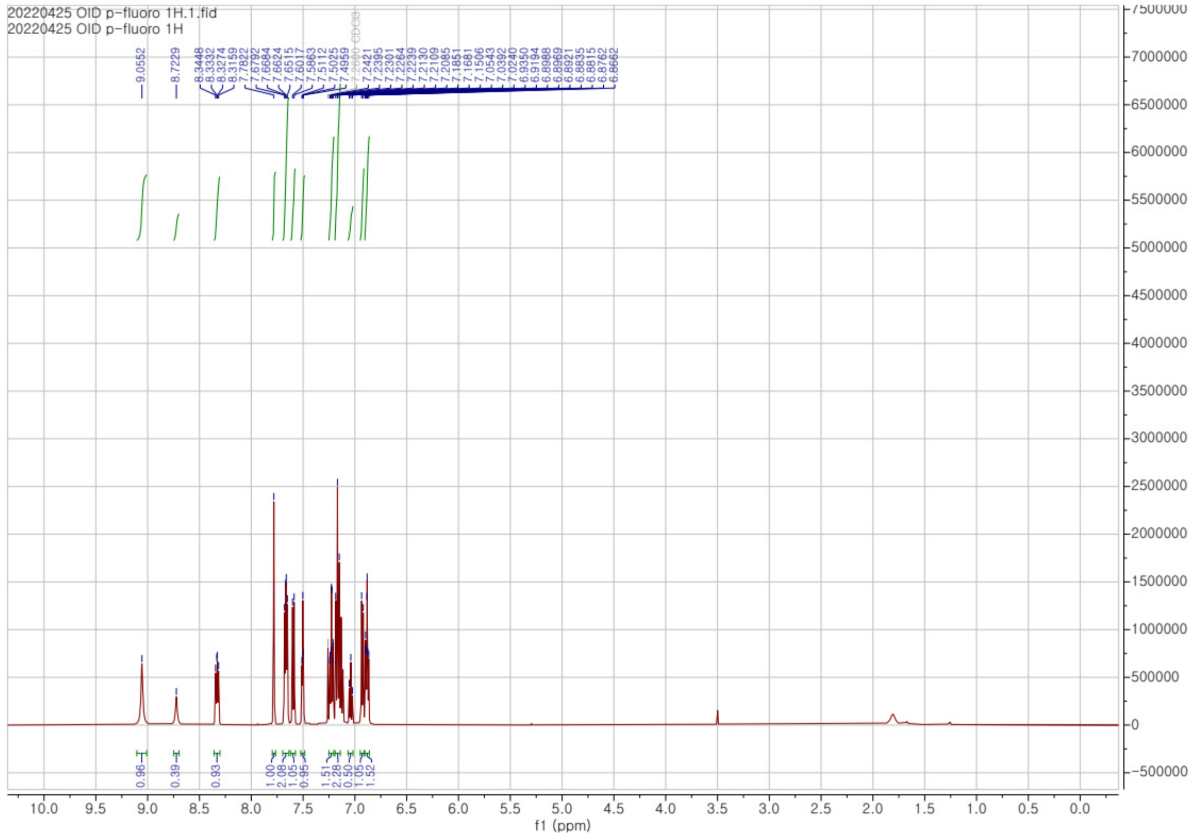

13C NMR of (Z)-3-(4-fluorobenzylidene) indolin-2-one

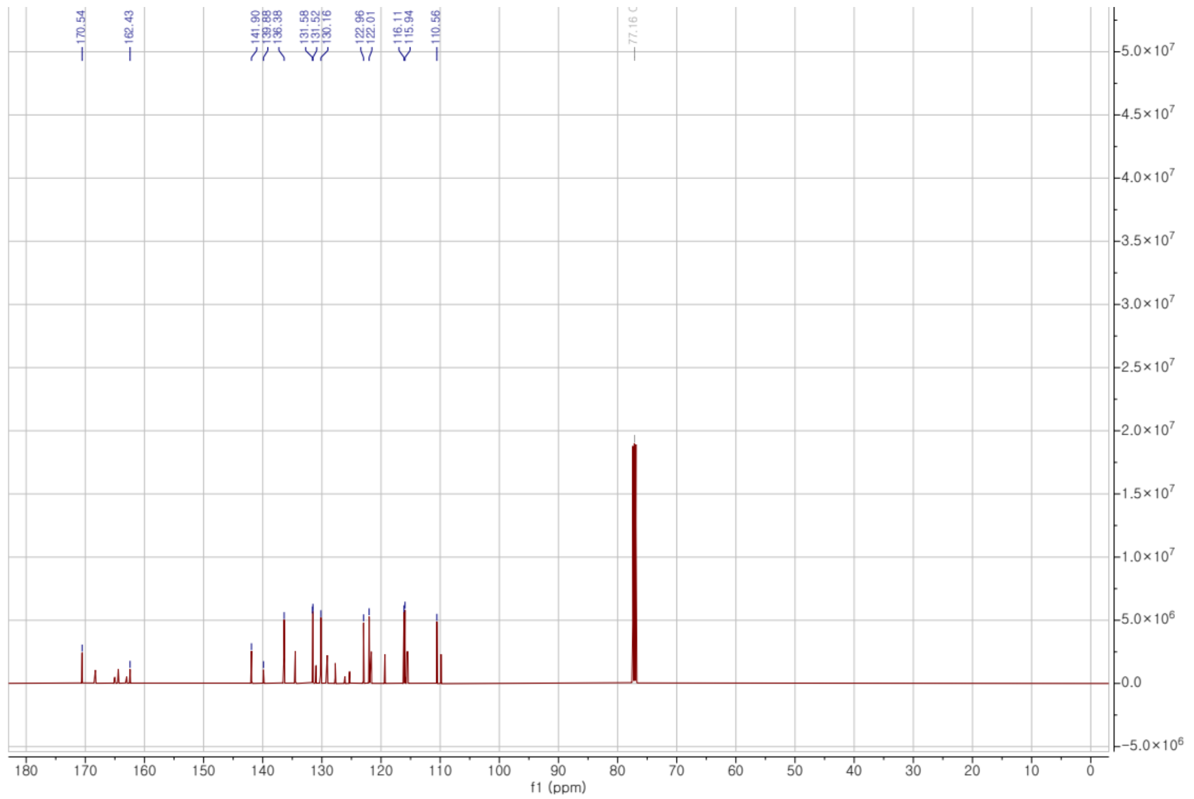

1H NMR of (Z)-3-(3-fluorobenzylidene) indolin-2-one

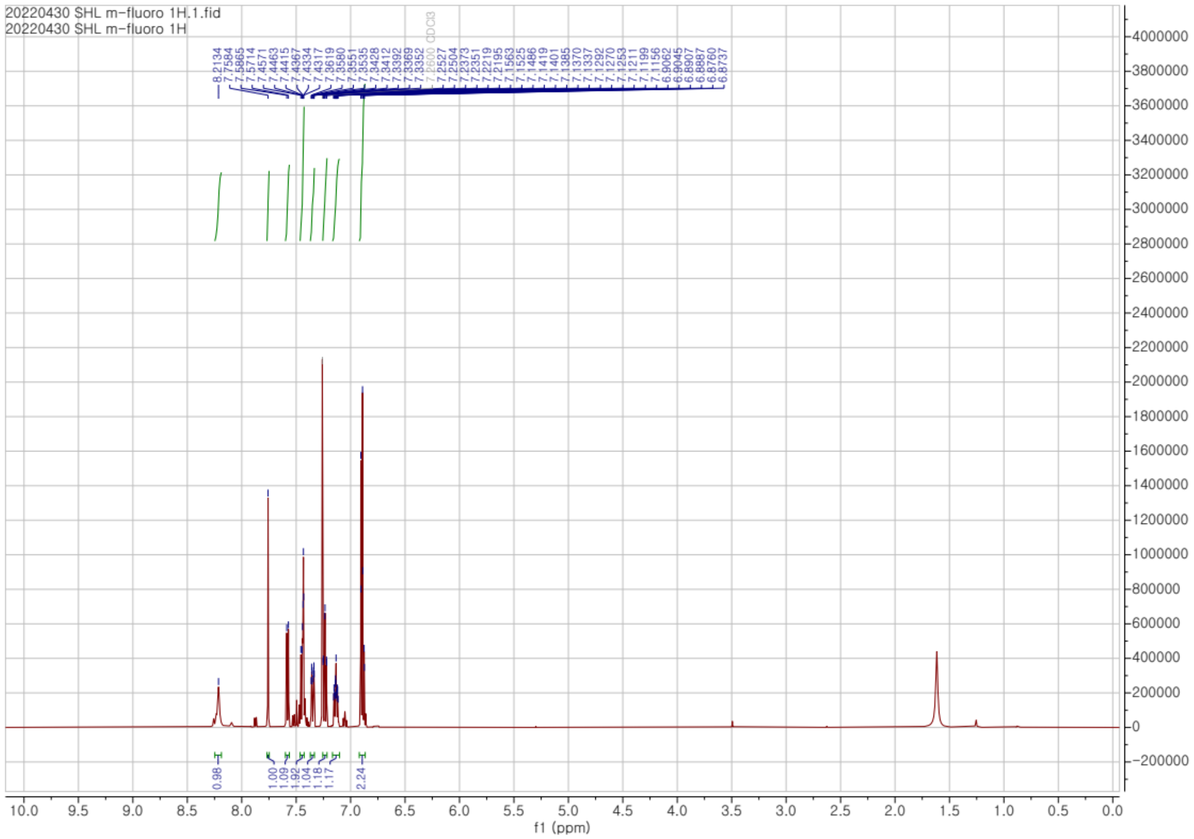

13C NMR of (Z)-3-(3-fluorobenzylidene) indolin-2-one

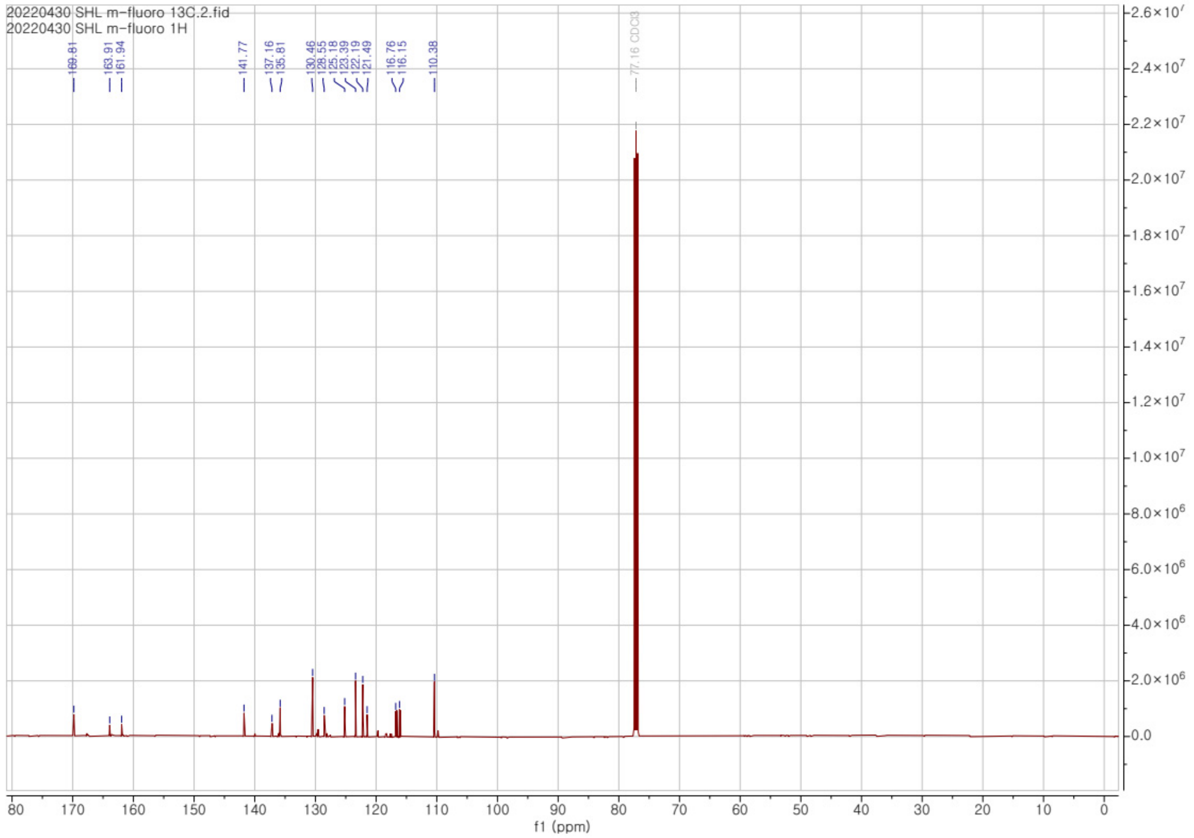

1H NMR of (Z)-3-(2-fluorobenzylidene) indolin-2-one

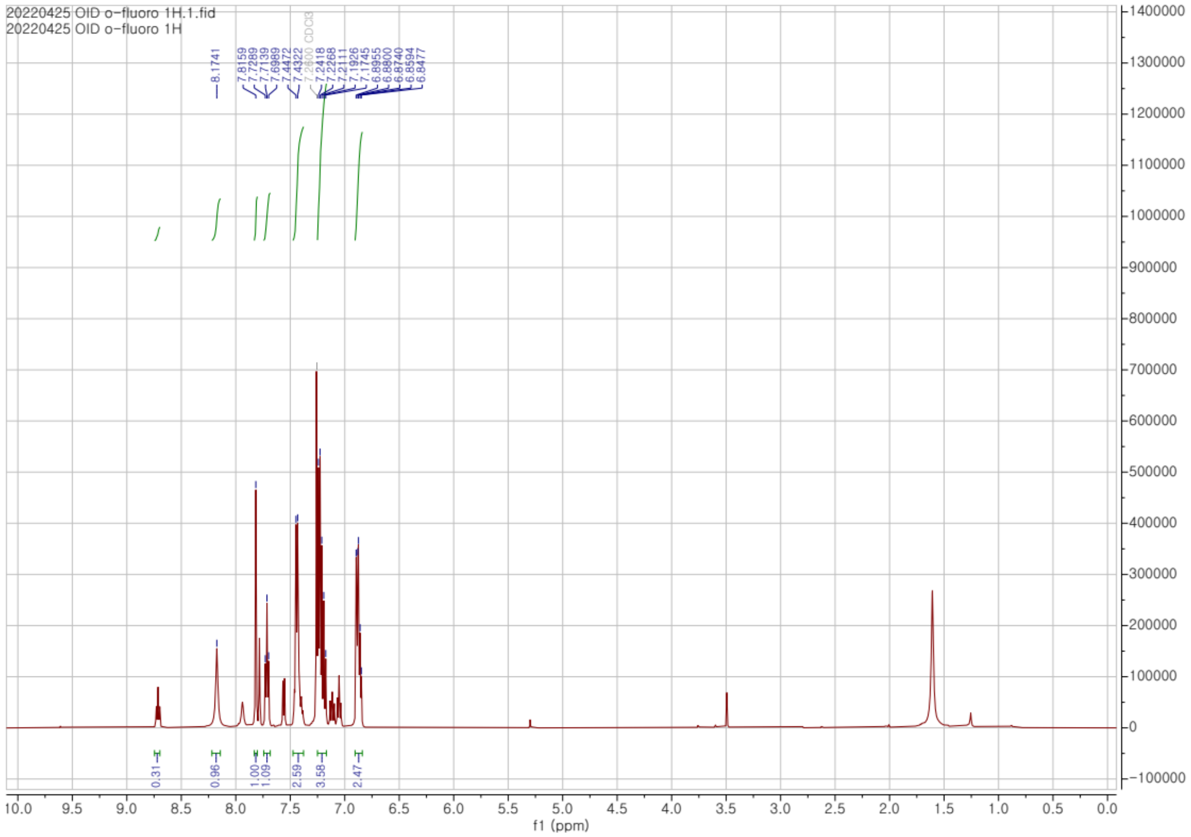

13C NMR of (Z)-3-(2-fluorobenzylidene) indolin-2-one

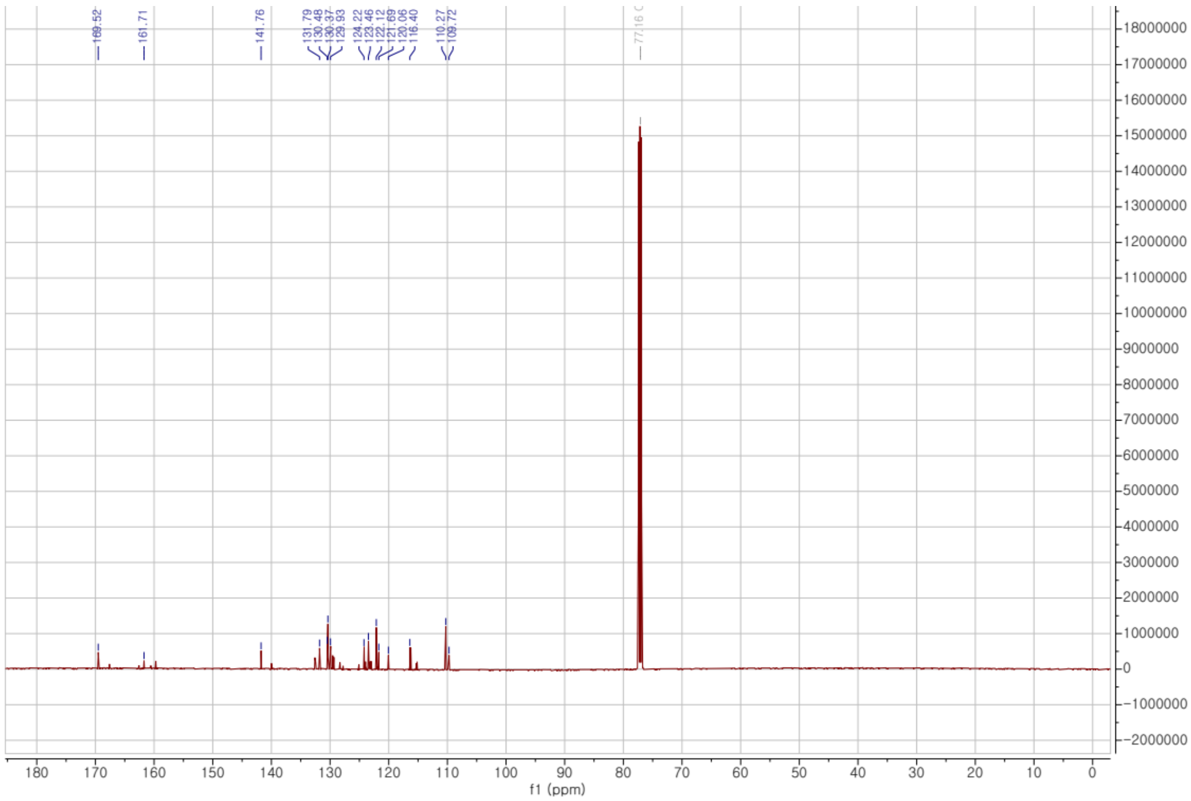

1H NMR of (Z)-3-(4-hydroxybenzylidene)indolin-2-one

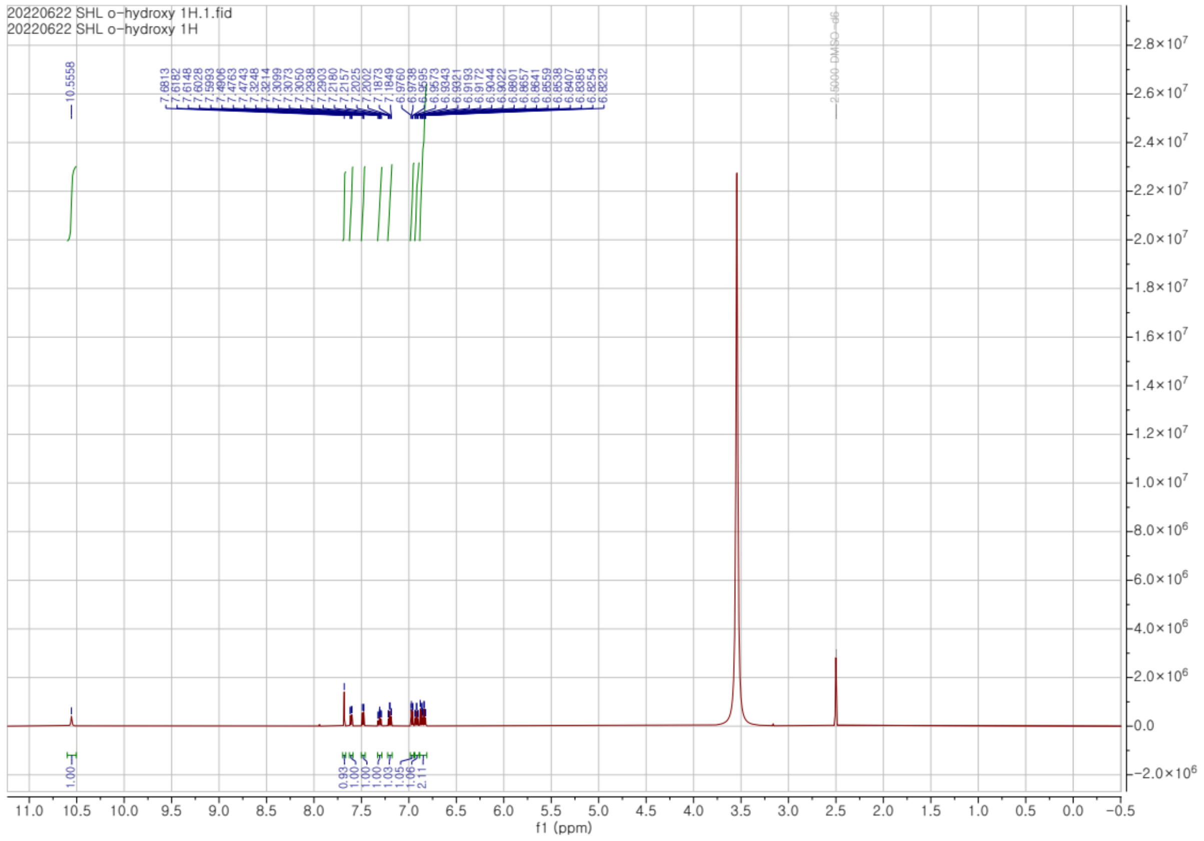

13C NMR of (Z)-3-(4-hydroxybenzylidene)indolin-2-one

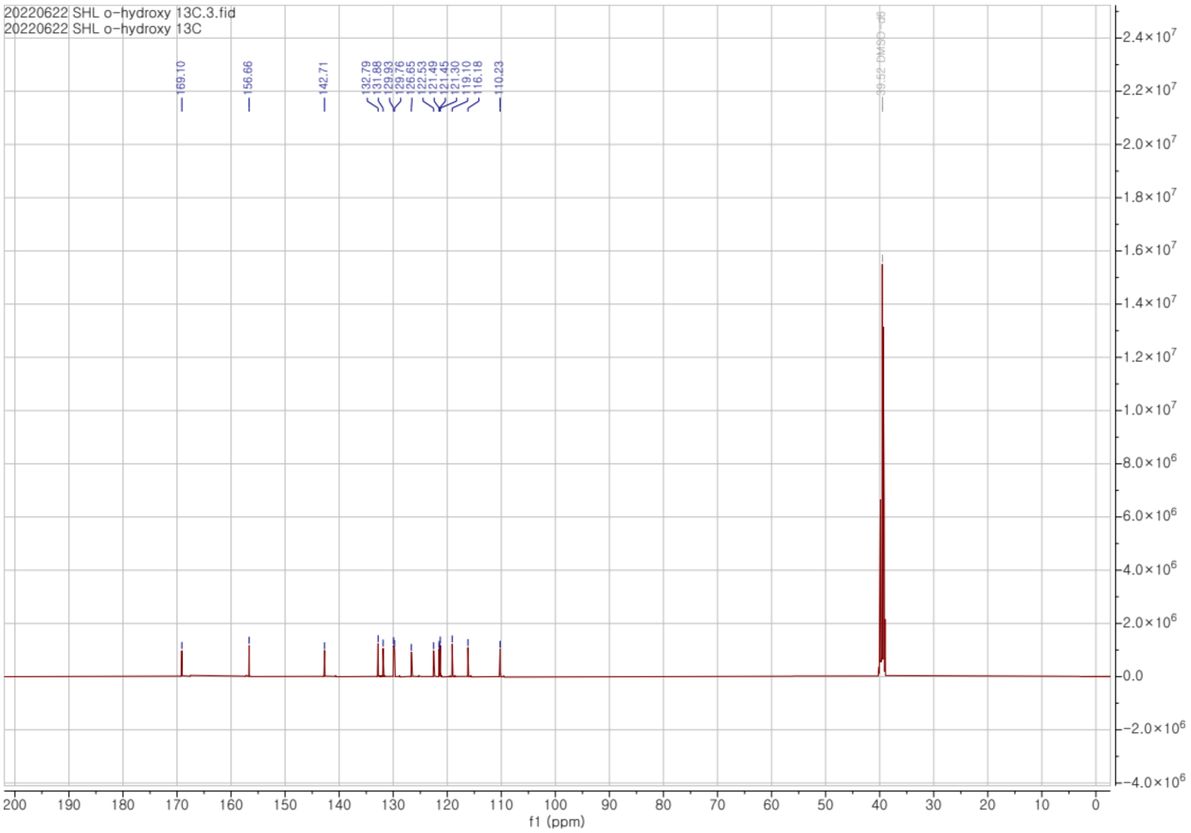

1H NMR of (Z)-3-(3-hydroxybenzylidene)indolin-2-one

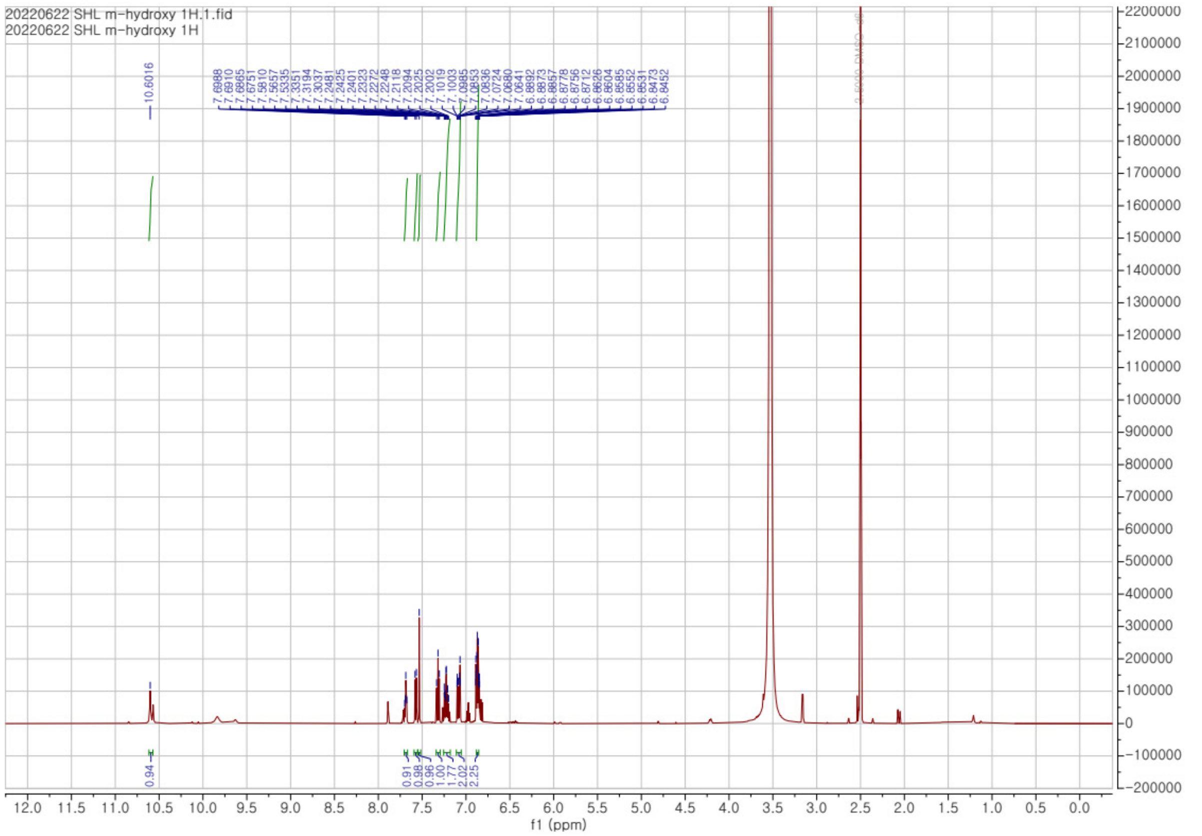

13C NMR of (Z)-3-(3-hydroxybenzylidene)indolin-2-one

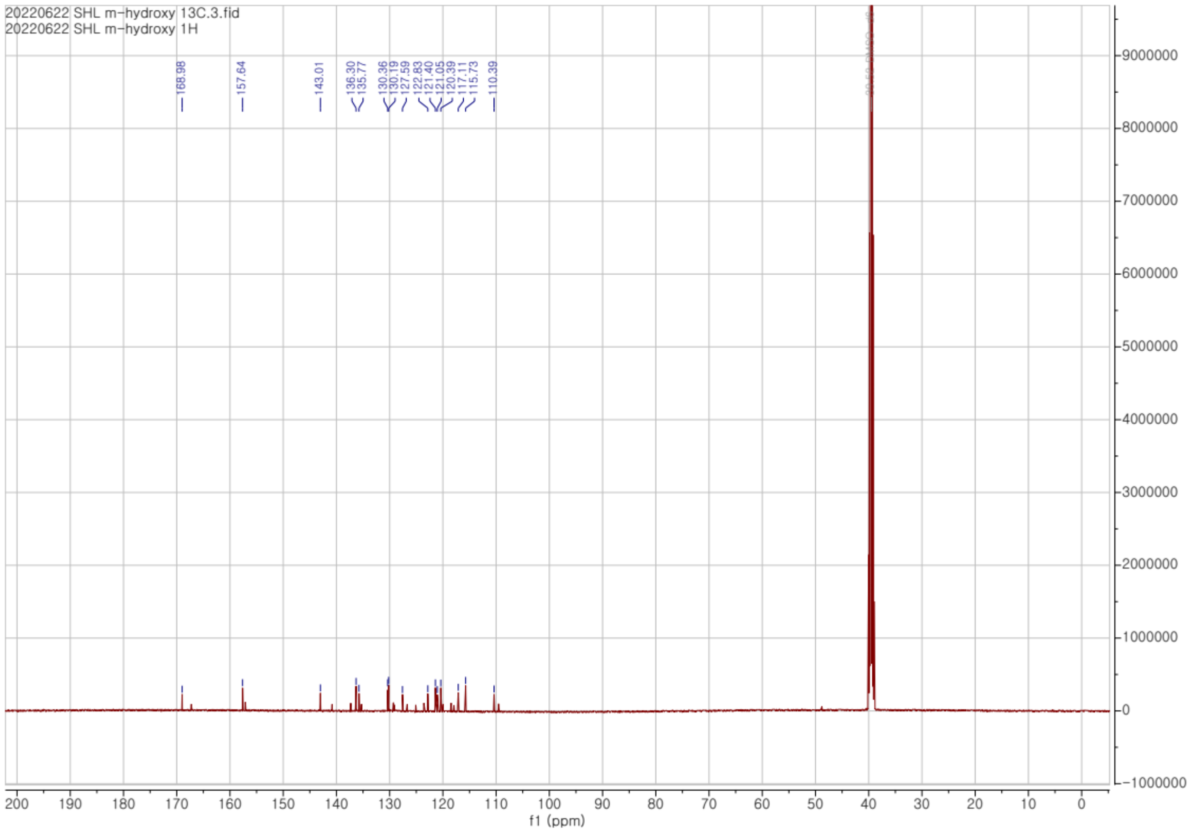

1H NMR of (Z)-3-(2-hydroxybenzylidene)indolin-2-one

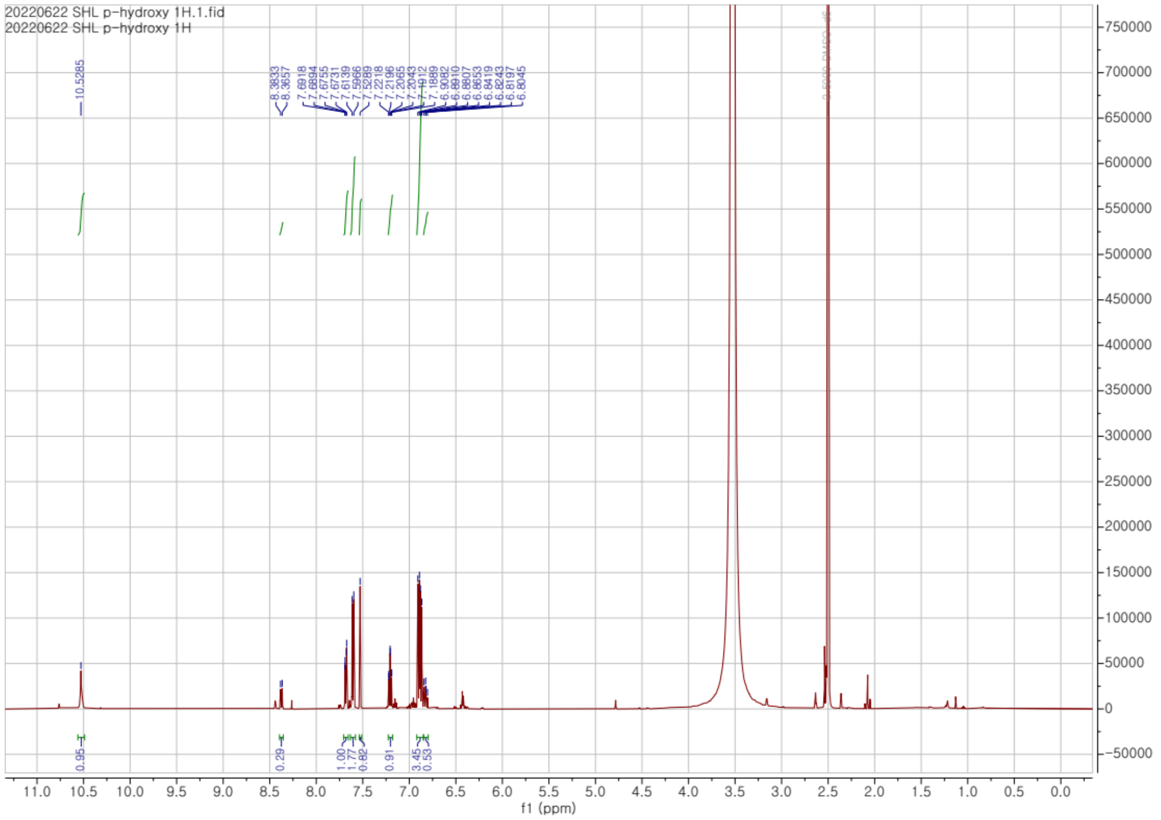

13C NMR of (Z)-3-(2-hydroxybenzylidene)indolin-2-one

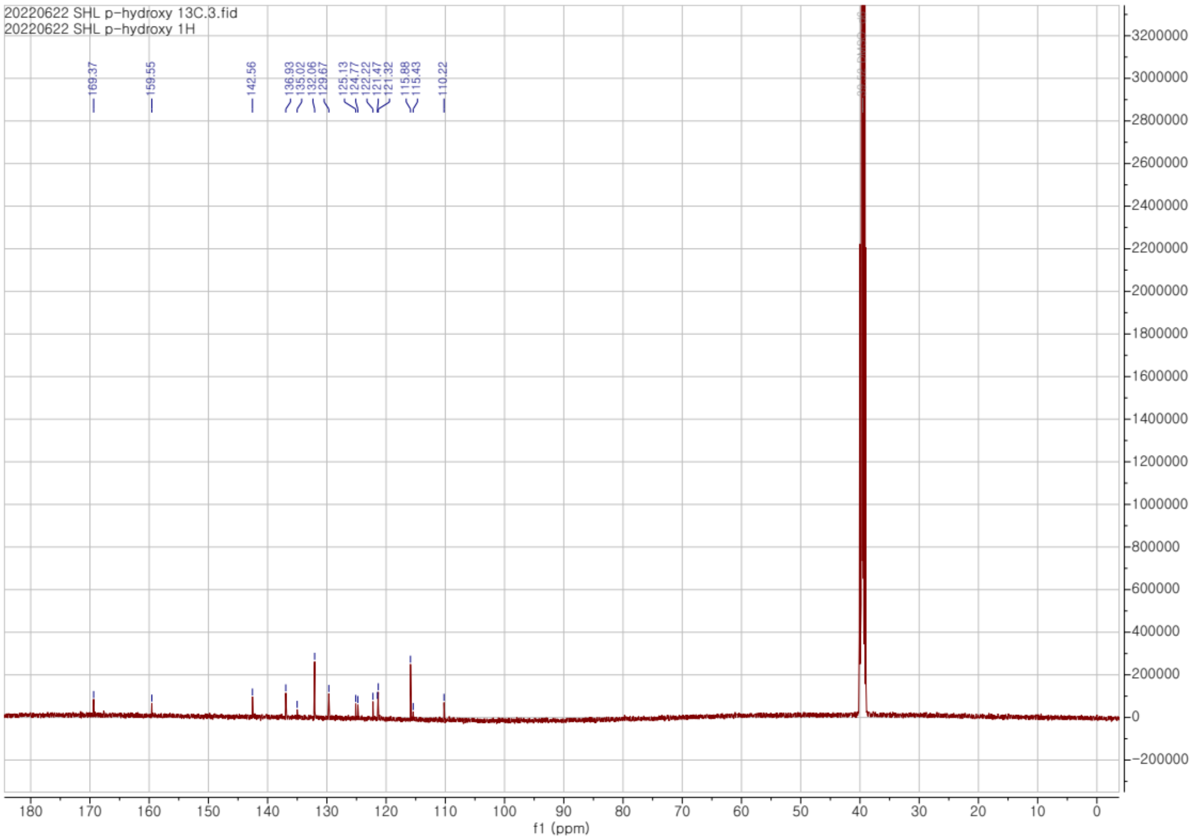

1H NMR of (Z)-3-(4-bromobenzylidene) indolin-2-one

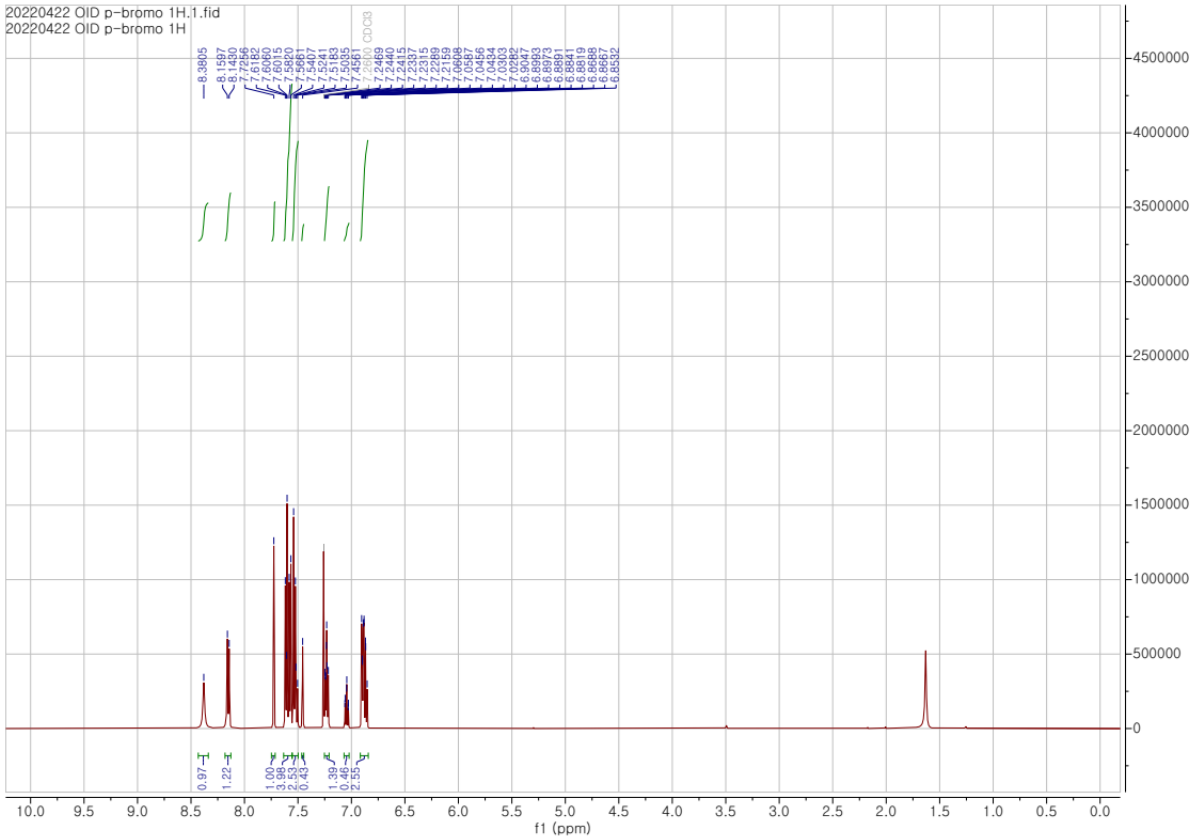

13C NMR of (Z)-3-(4-bromobenzylidene) indolin-2-one

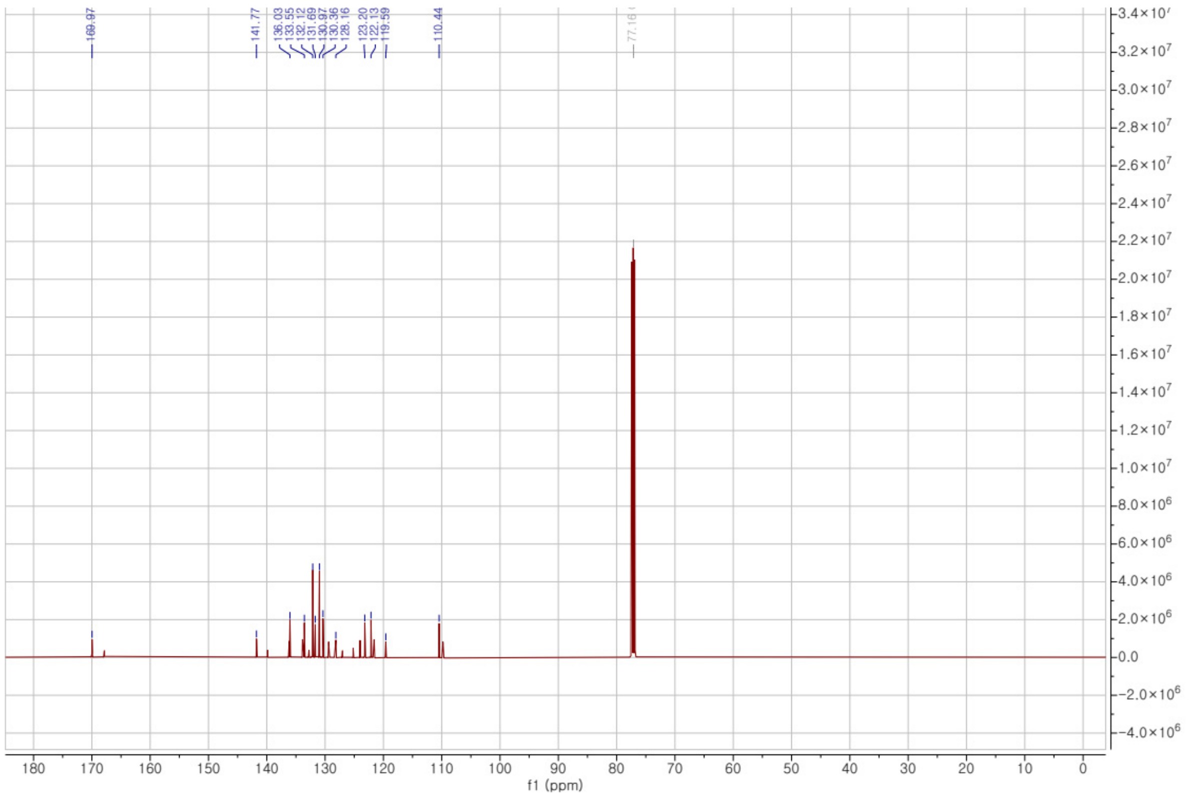

1H NMR of (Z)-3-(3-bromobenzylidene) indolin-2-one

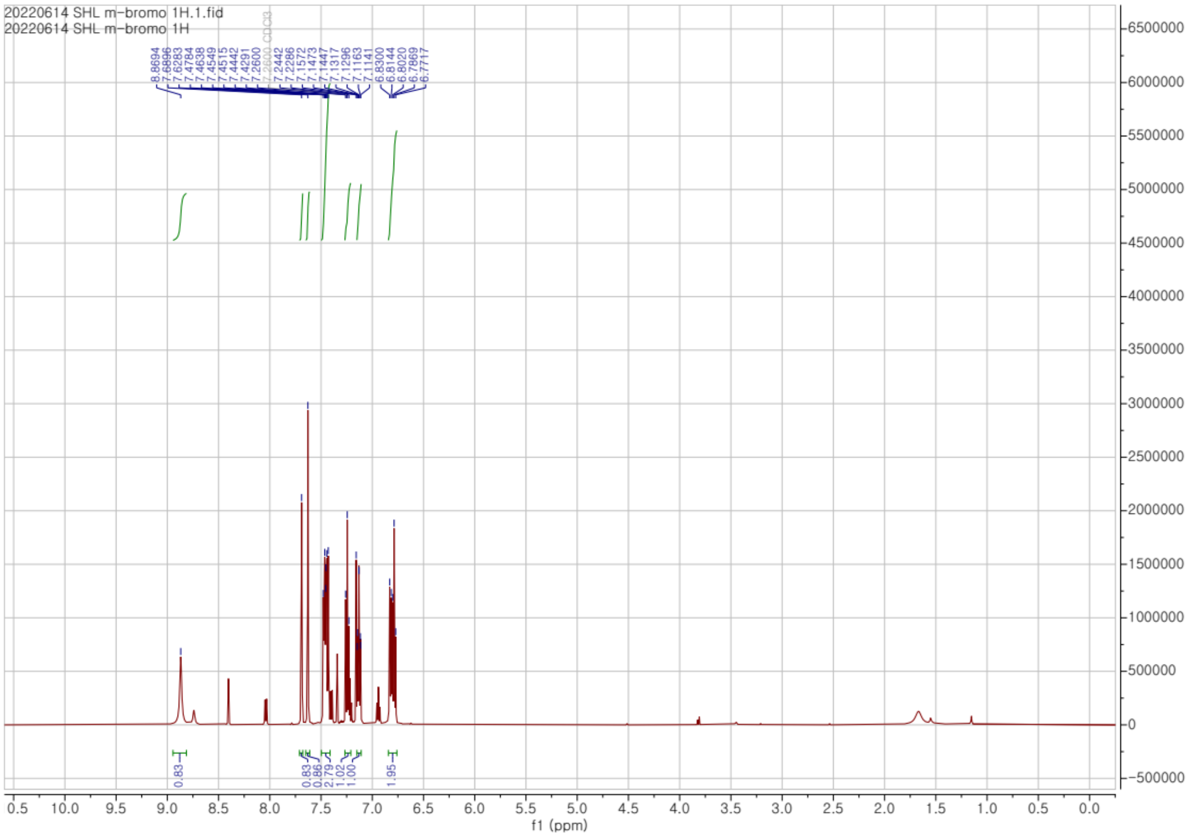

13C NMR of (Z)-3-(3-bromobenzylidene) indolin-2-one

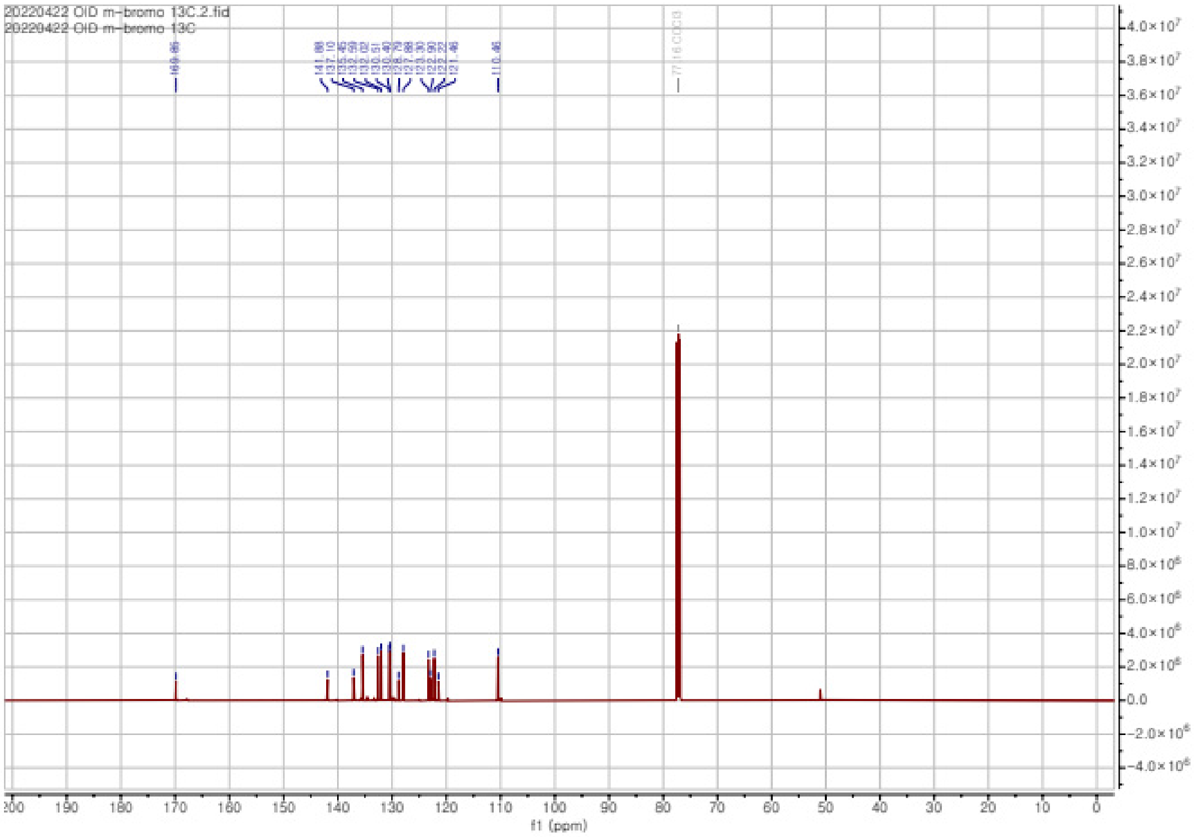

1H NMR of (Z)-3-(2-bromobenzylidene) indolin-2-one

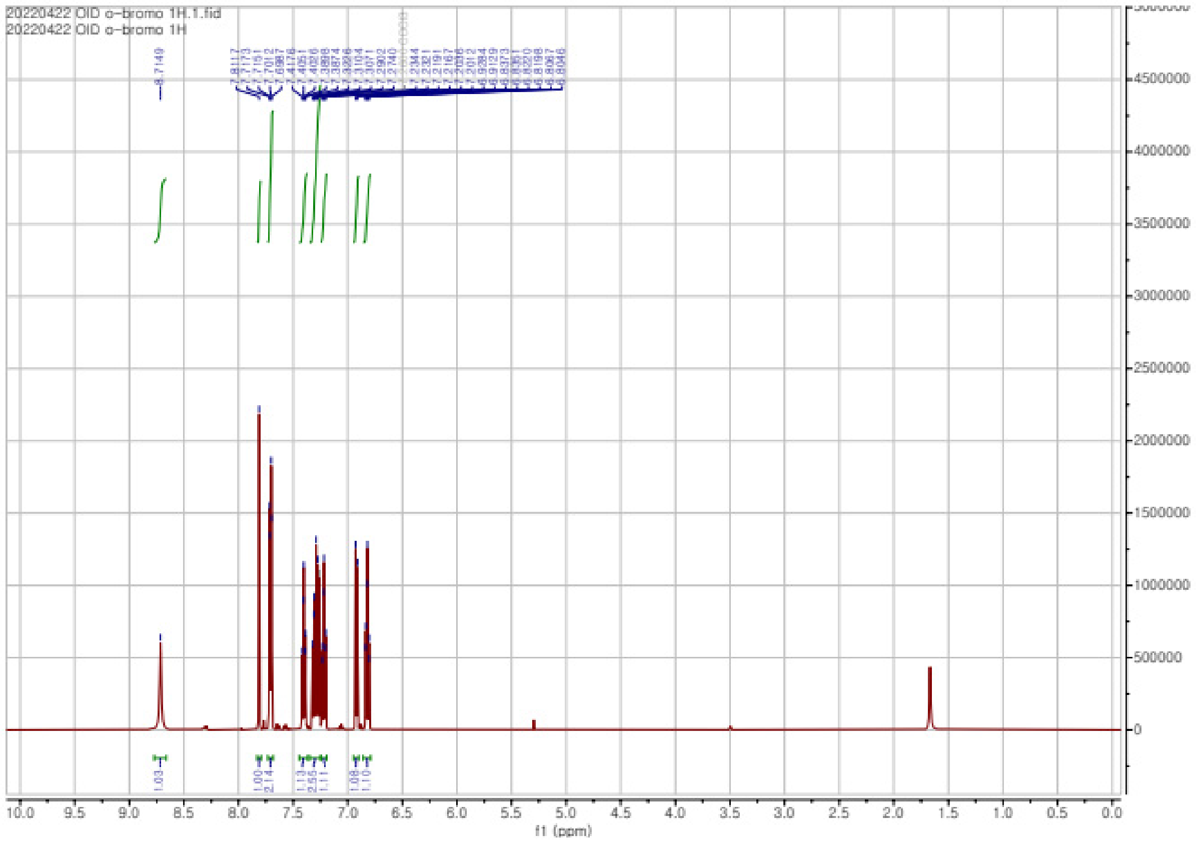

13C NMR of (Z)-3-(2-bromobenzylidene) indolin-2-one

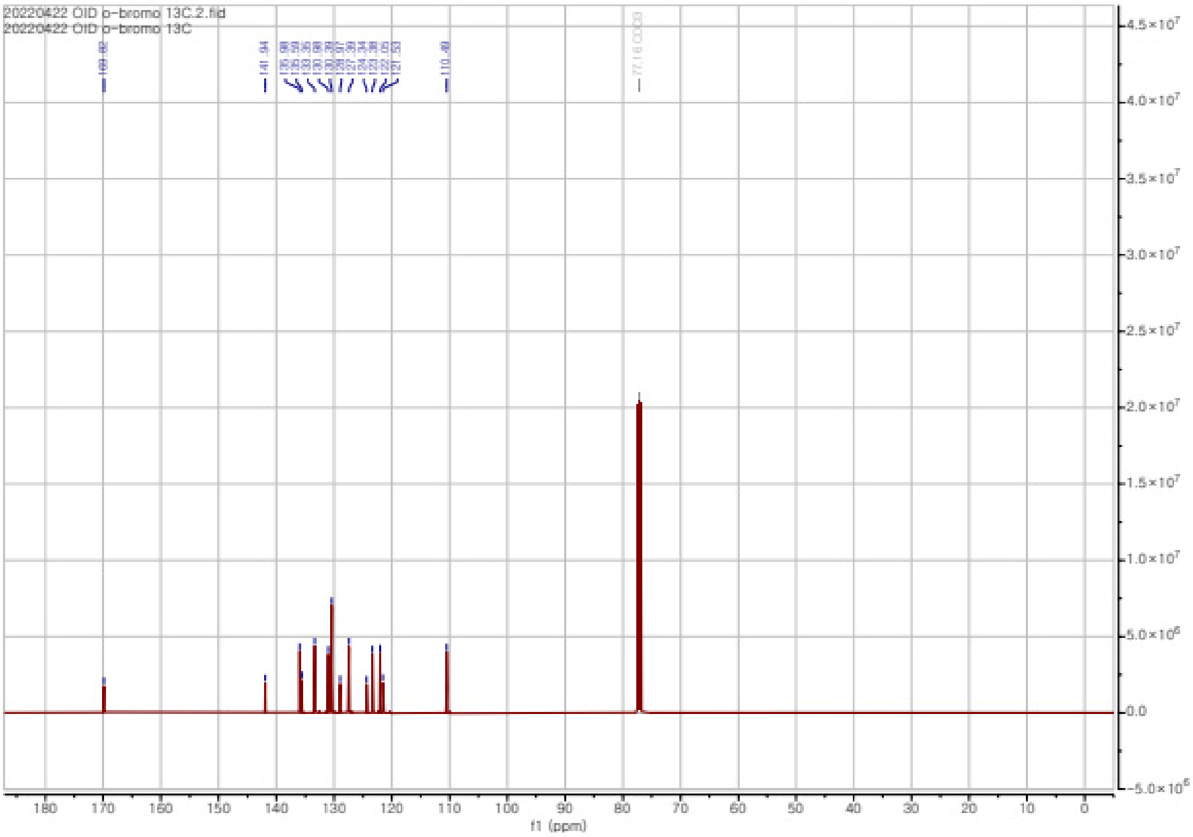

1H NMR of (Z)-4-((2-oxoindolin-3-ylidene) methyl) benzonitrile

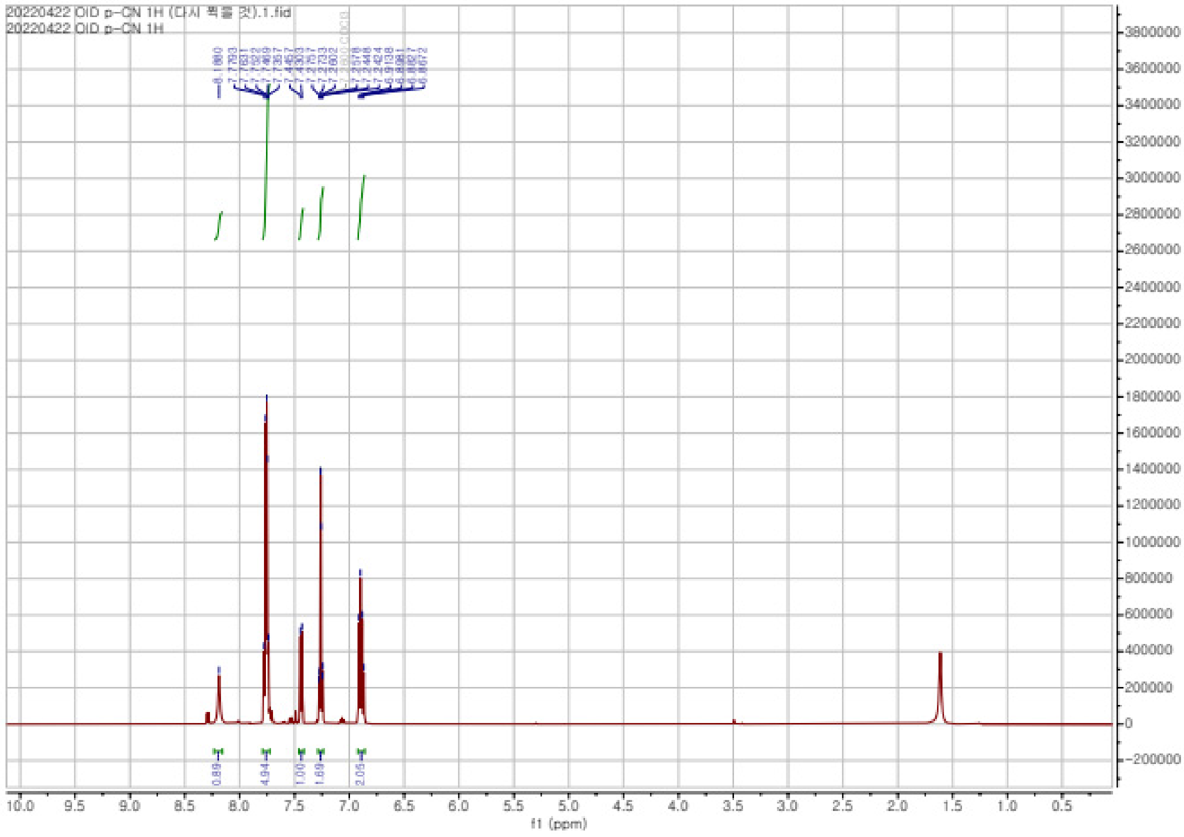

13C NMR of (Z)-4-((2-oxoindolin-3-ylidene) methyl) benzonitrile

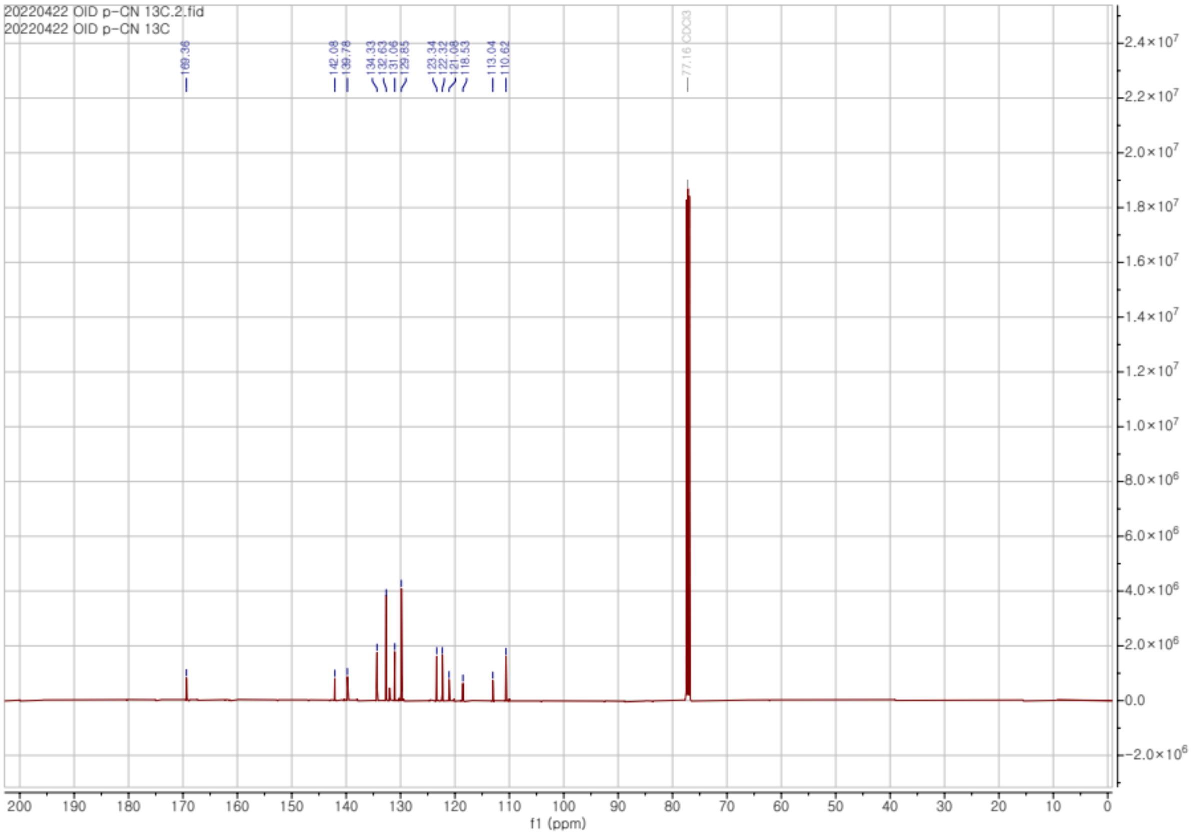

1H NMR of (Z)-3-(4-(trifluoromethyl) benzylidene) indolin-2-one

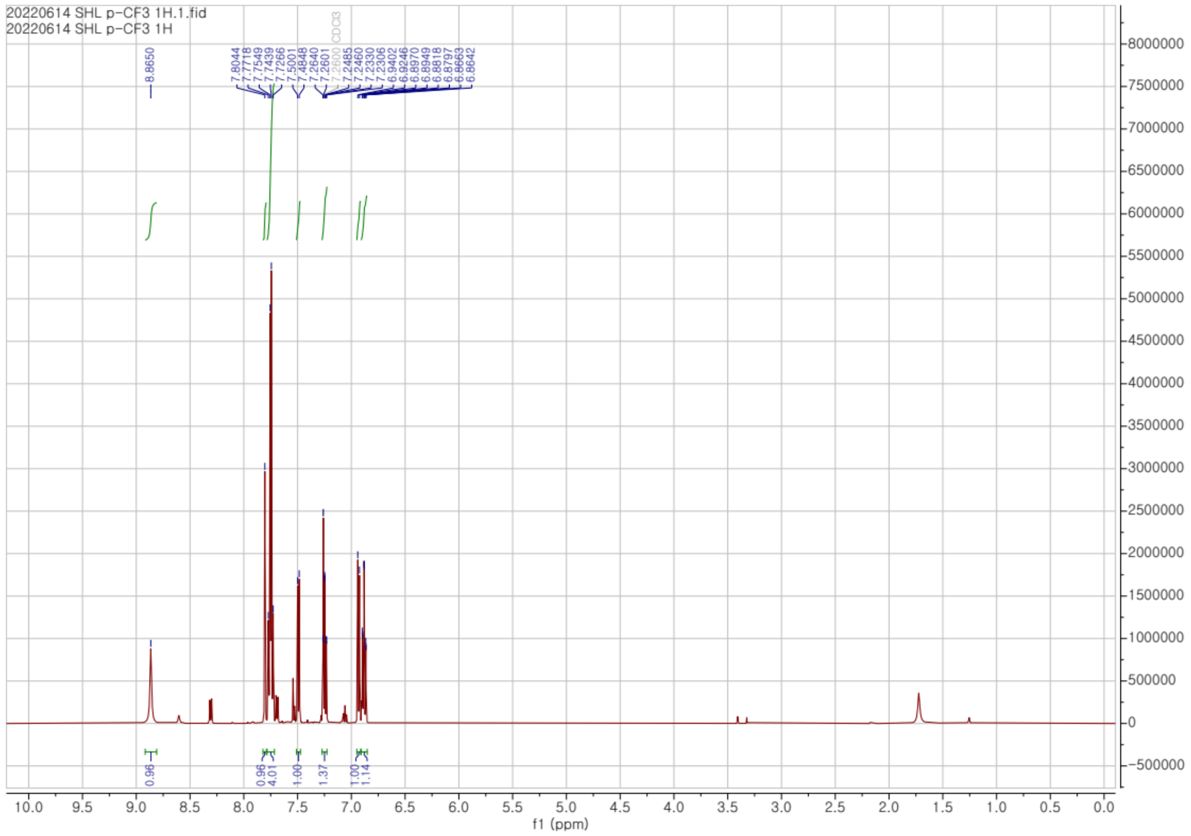

13C NMR of (Z)-3-(4-(trifluoromethyl) benzylidene) indolin-2-one

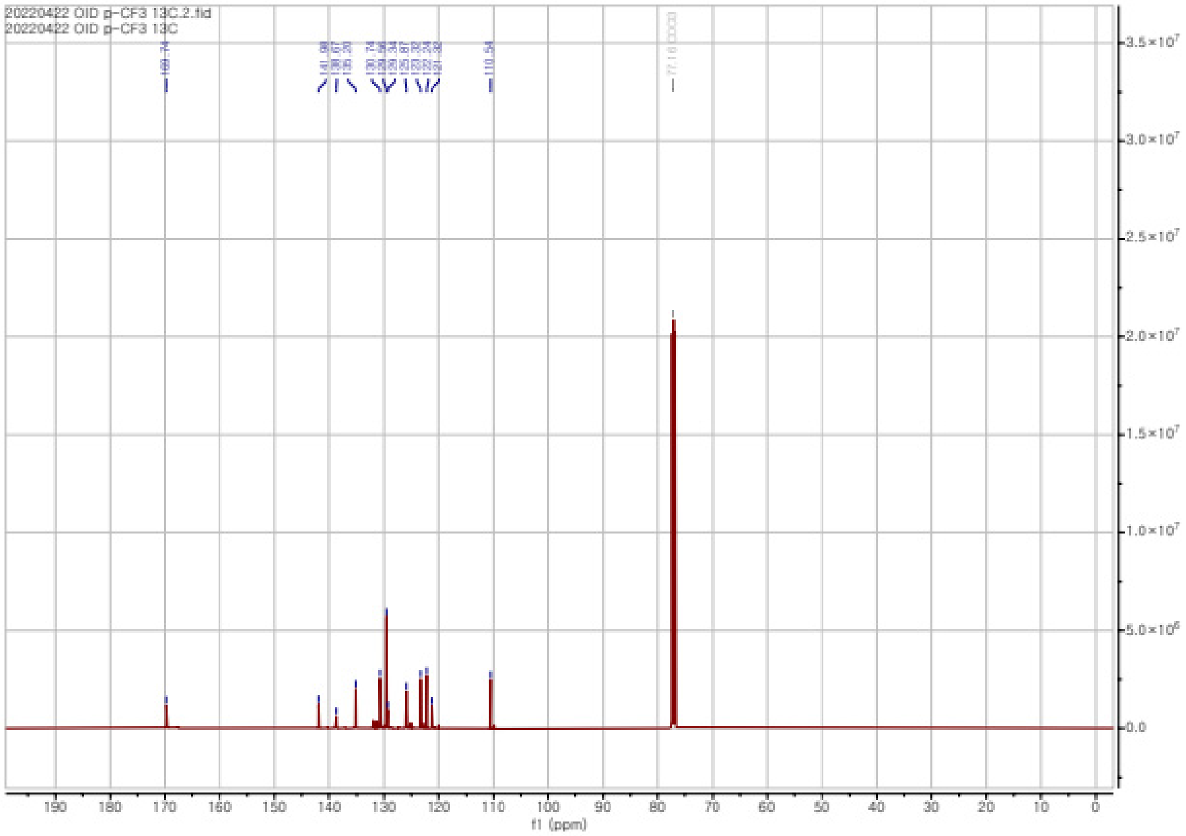

1H NMR of (Z)-3-(3-(trifluoromethyl) benzylidene) indolin-2-one

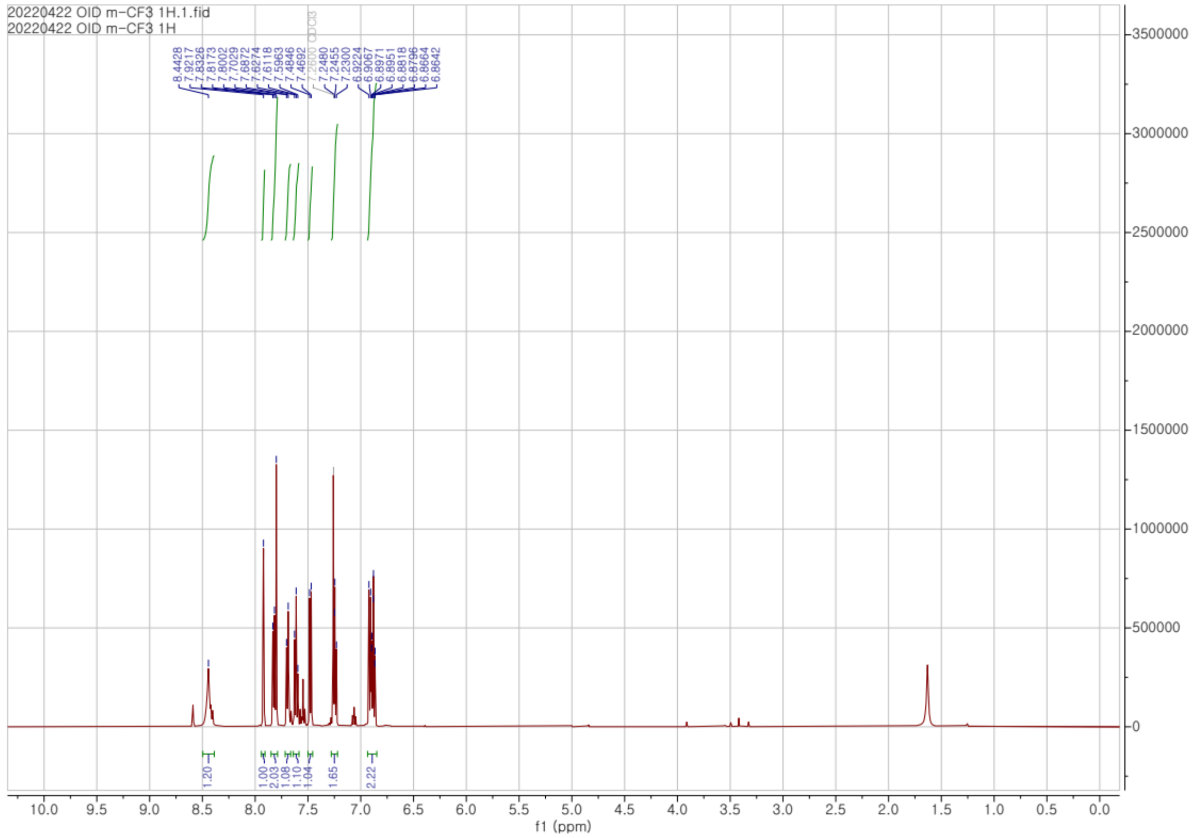

13C NMR of (Z)-3-(3-(trifluoromethyl) benzylidene) indolin-2-one

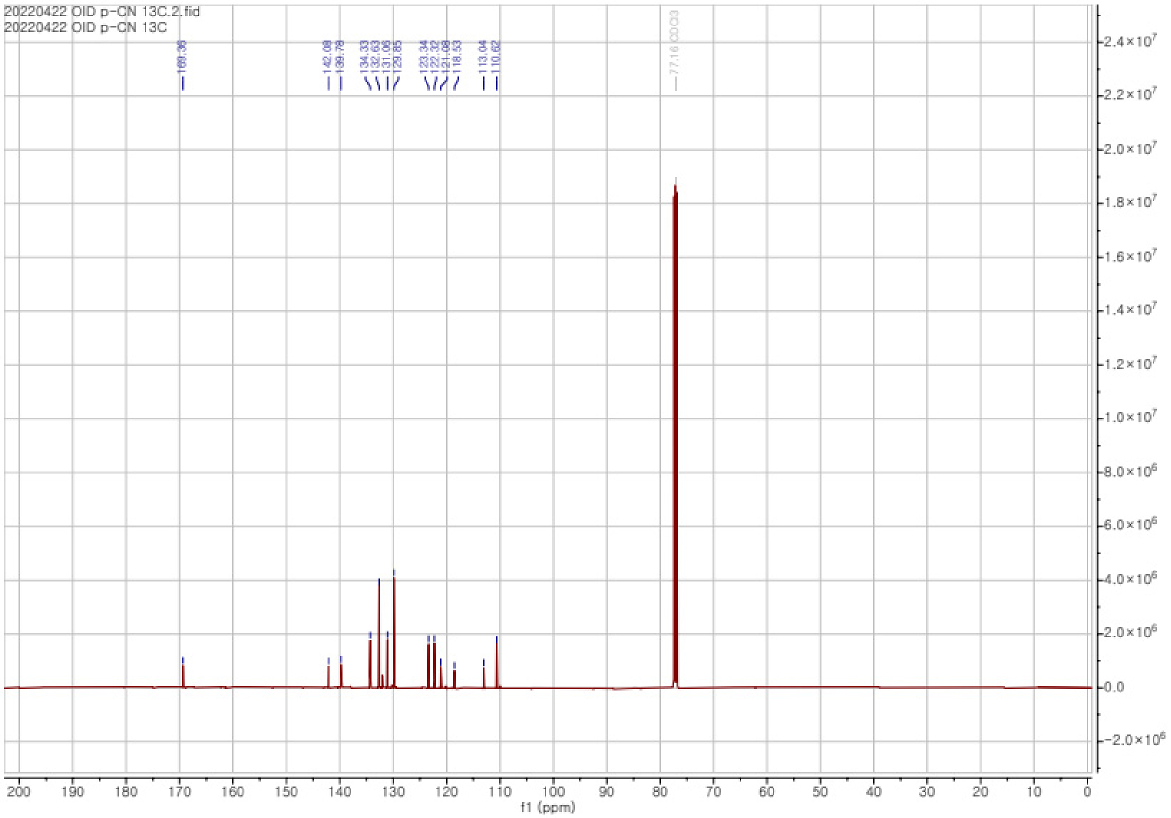

1H NMR of (Z)-3-(2-(trifluoromethyl) benzylidene) indolin-2-one

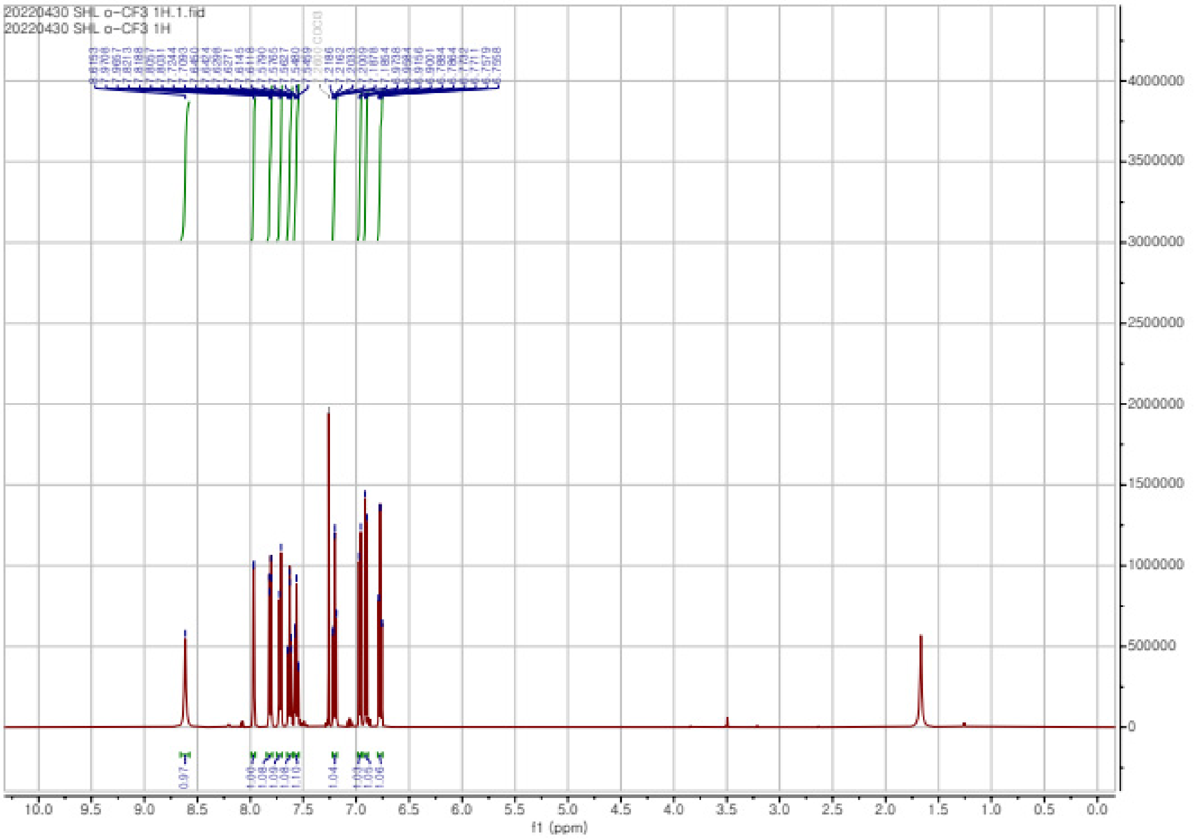

13C NMR of (Z)-3-(2-(trifluoromethyl) benzylidene) indolin-2-one

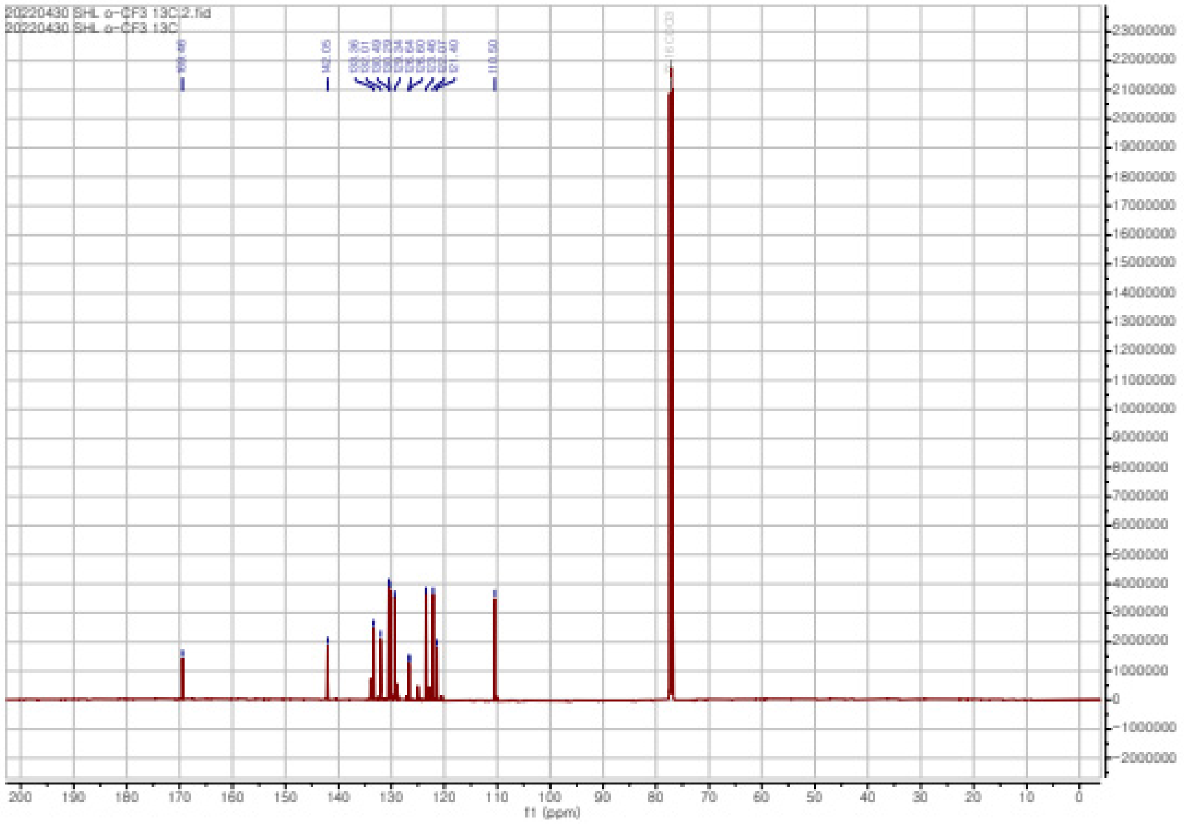

1H NMR of (Z)-3-(4-chlorobenzylidene) indolin-2-one

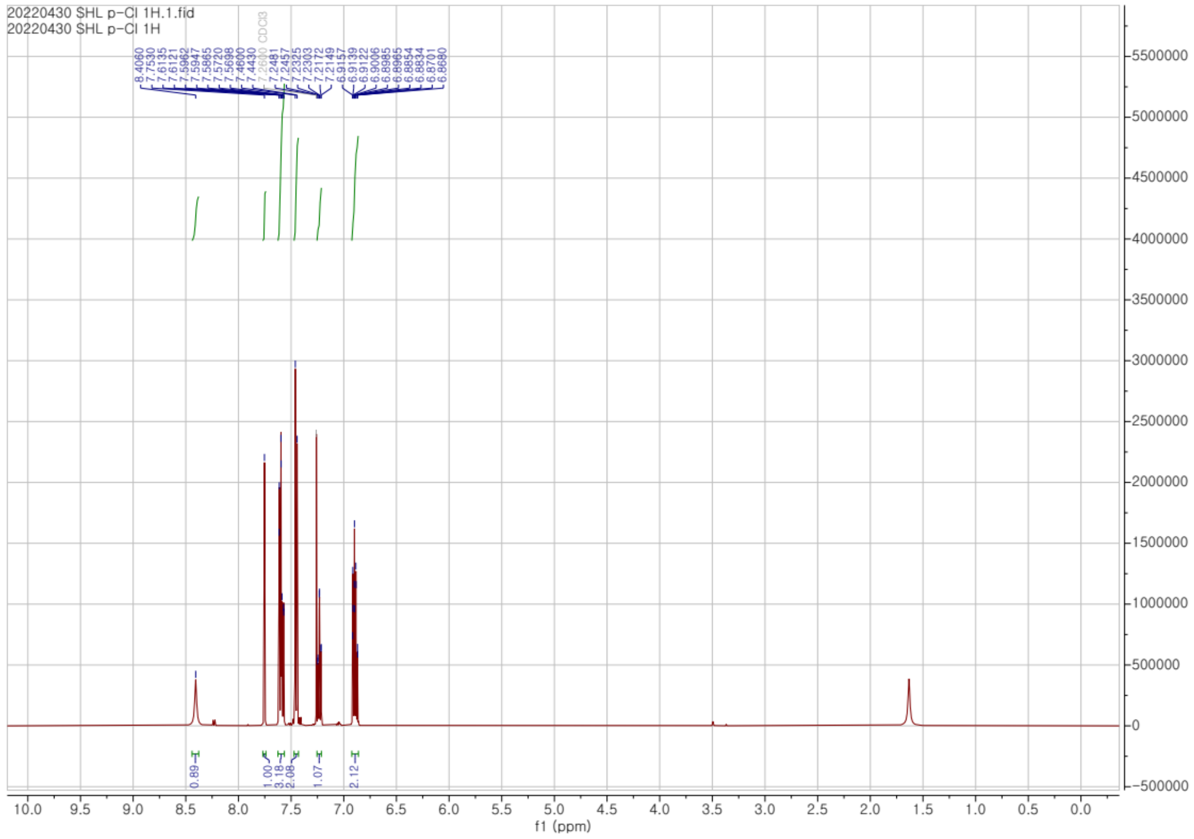

13C NMR of (Z)-3-(4-chlorobenzylidene) indolin-2-one

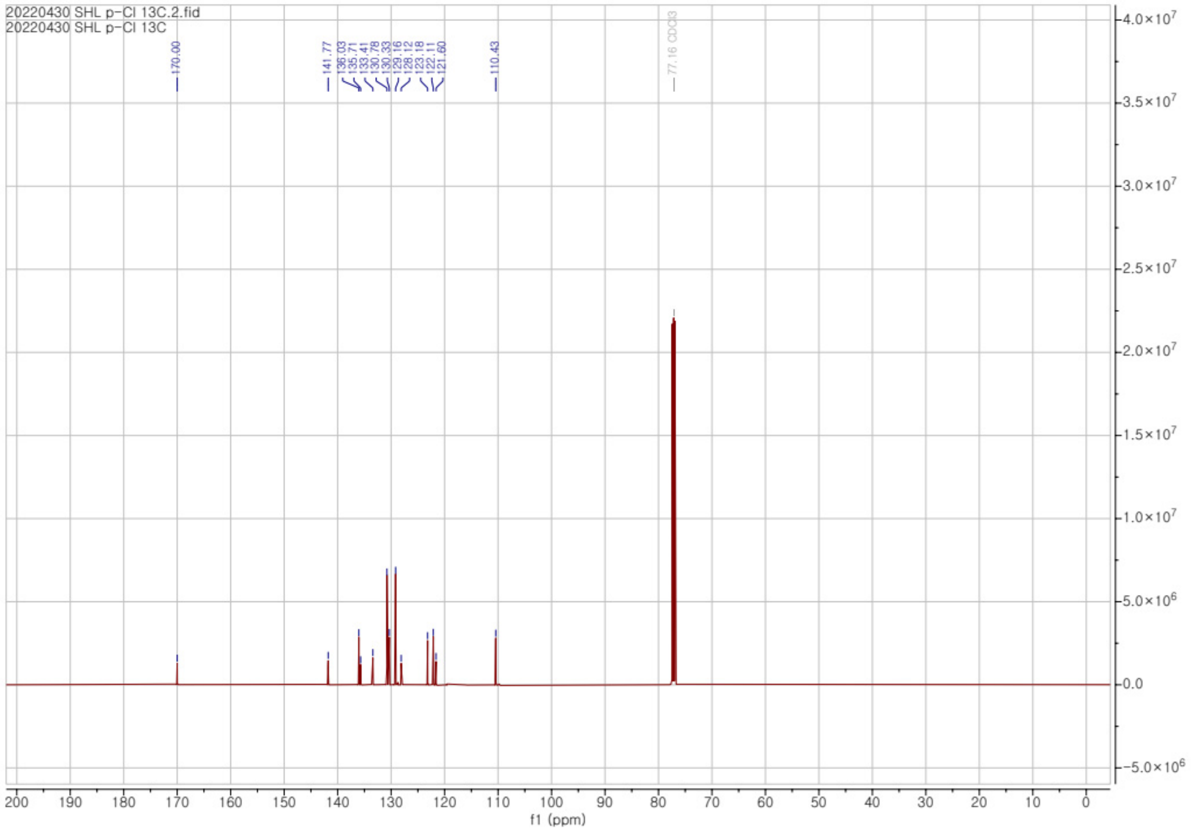

1H NMR of (Z)-3-(3-chlorobenzylidene) indolin-2-one

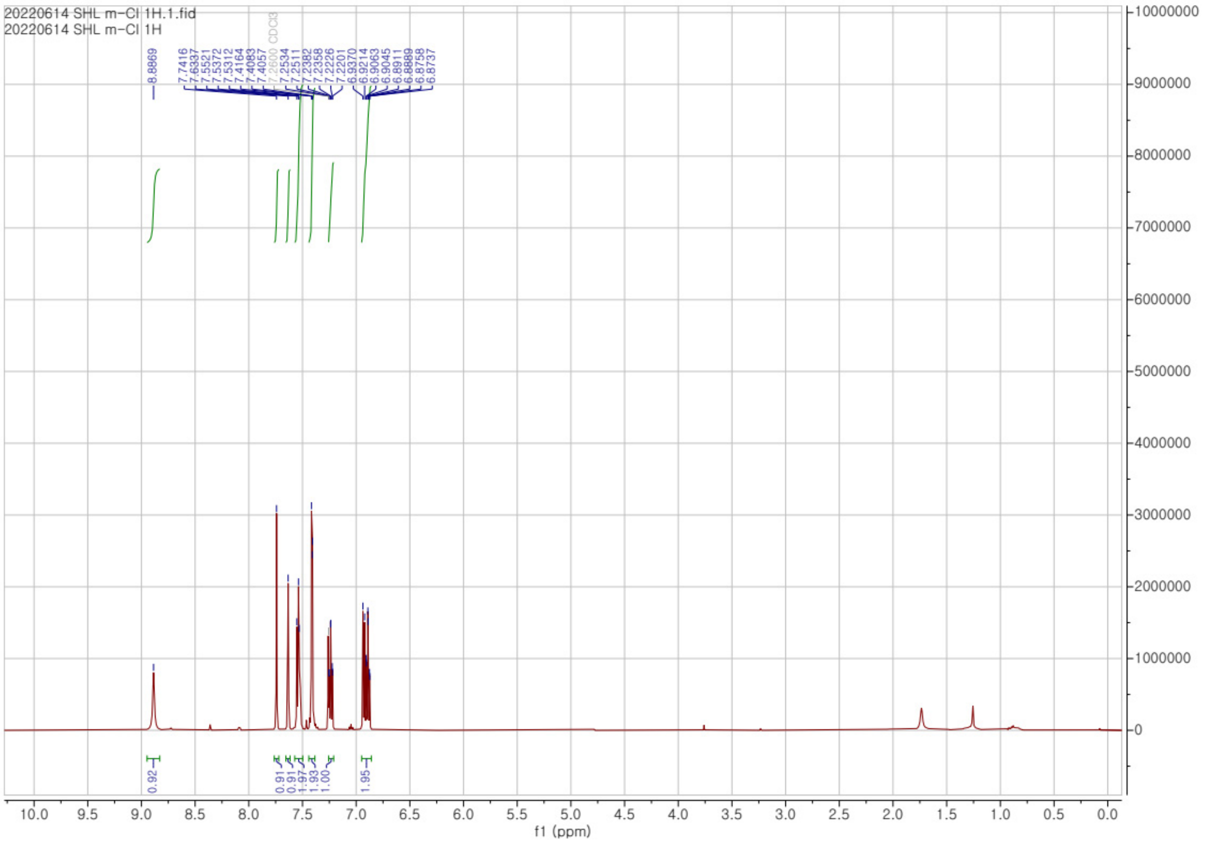

13C NMR of (Z)-3-(3-chlorobenzylidene) indolin-2-one

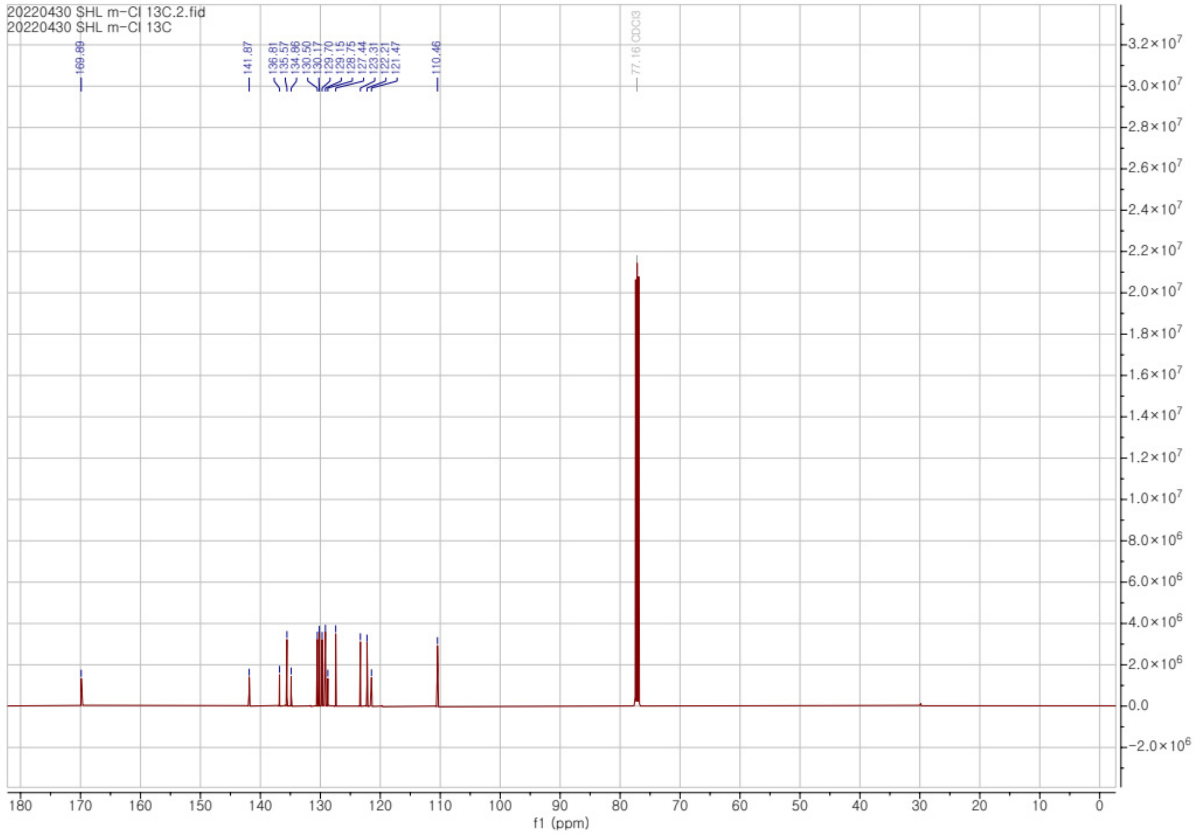

1H NMR of (Z)-3-(2-chlorobenzylidene) indolin-2-one

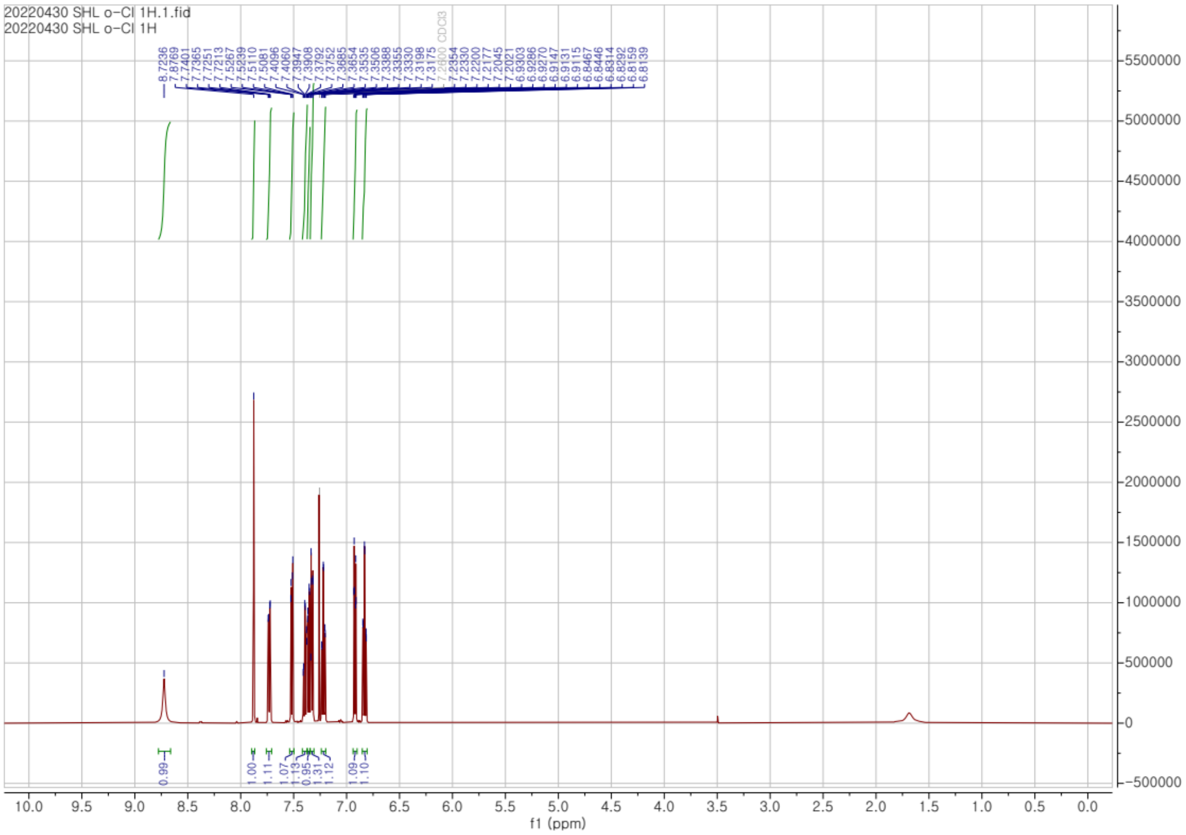

13C NMR of (Z)-3-(2-chlorobenzylidene) indolin-2-one

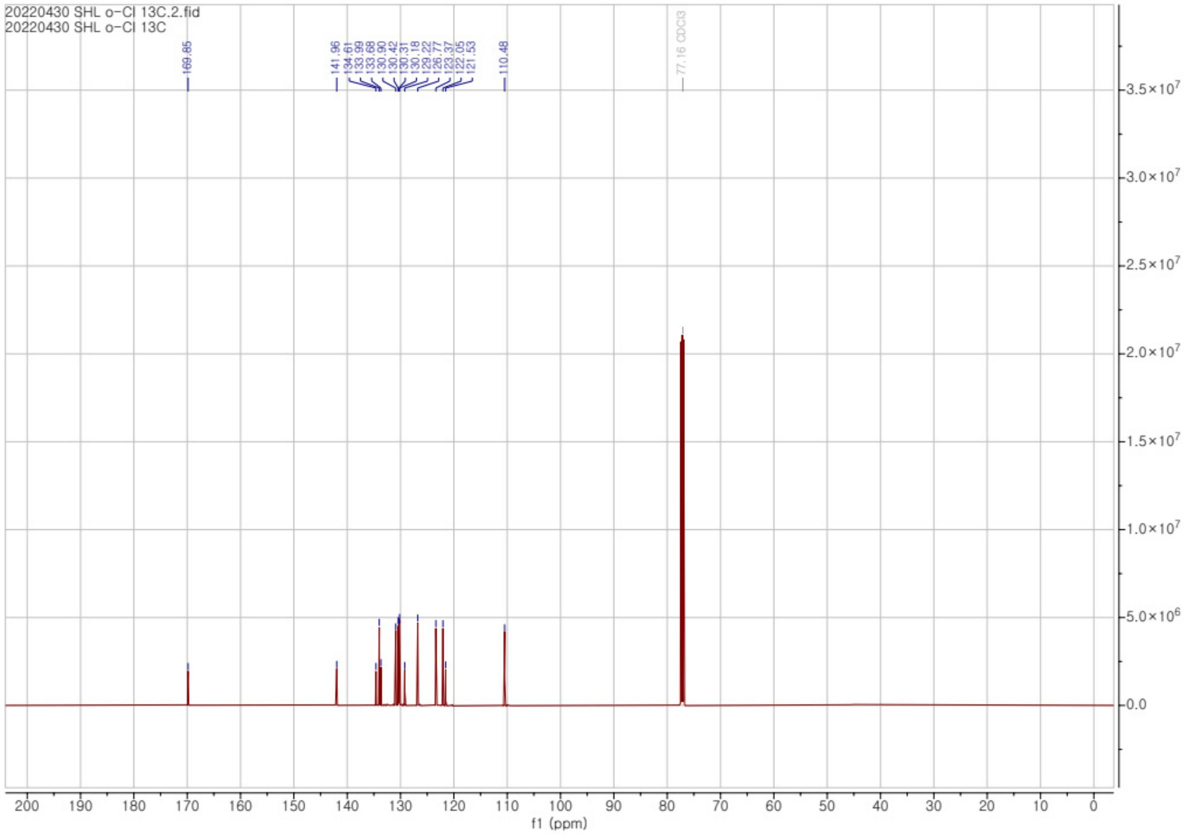

1H NMR of (Z)-3-(2-methoxybenzylidene)indolin-2-one

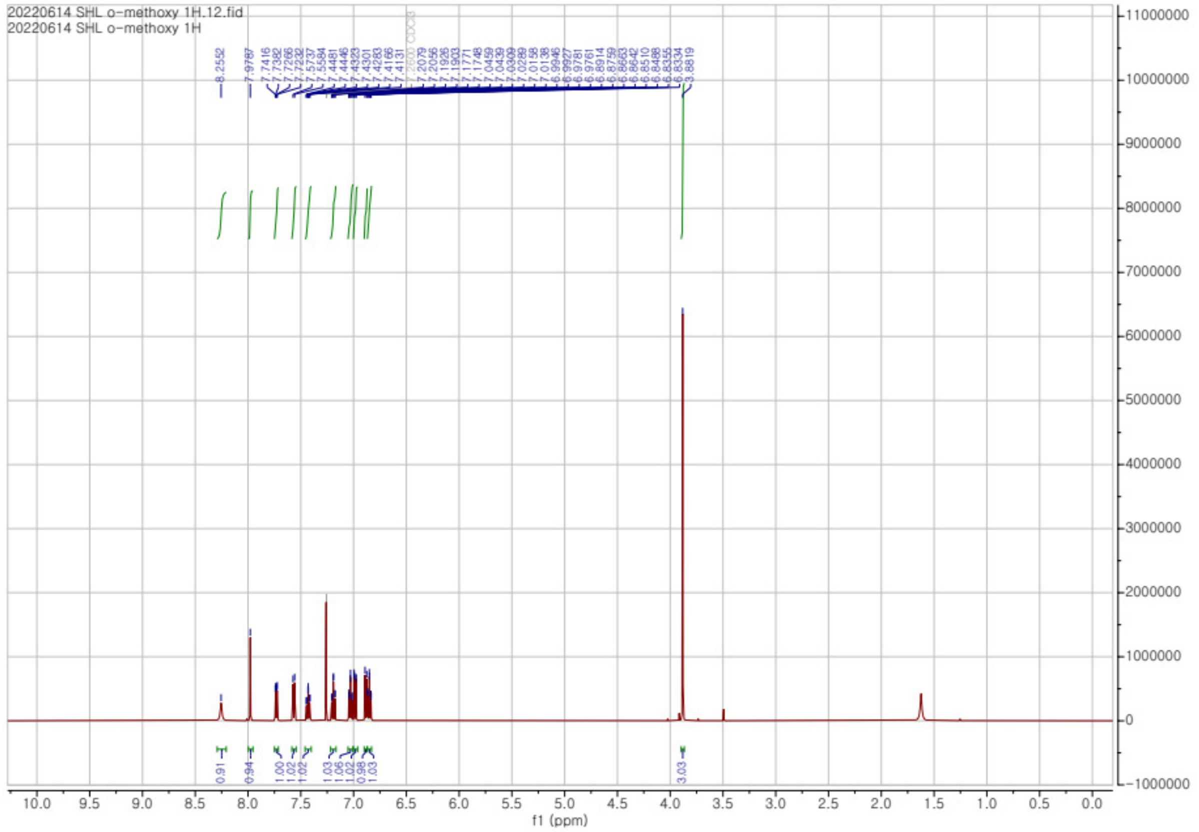

13C NMR of (Z)-3-(2-methoxybenzylidene)indolin-2-one

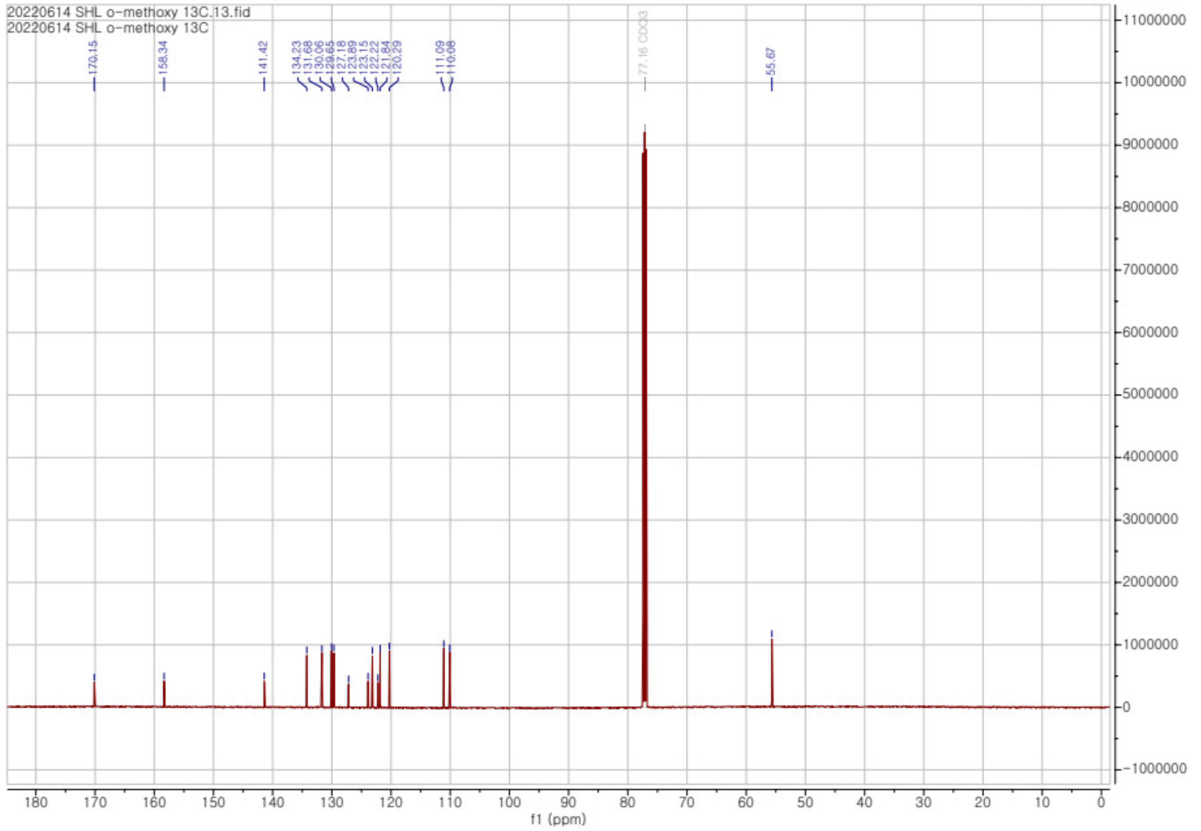

1H NMR of (Z)-3-(3-methoxybenzylidene)indolin-2-one

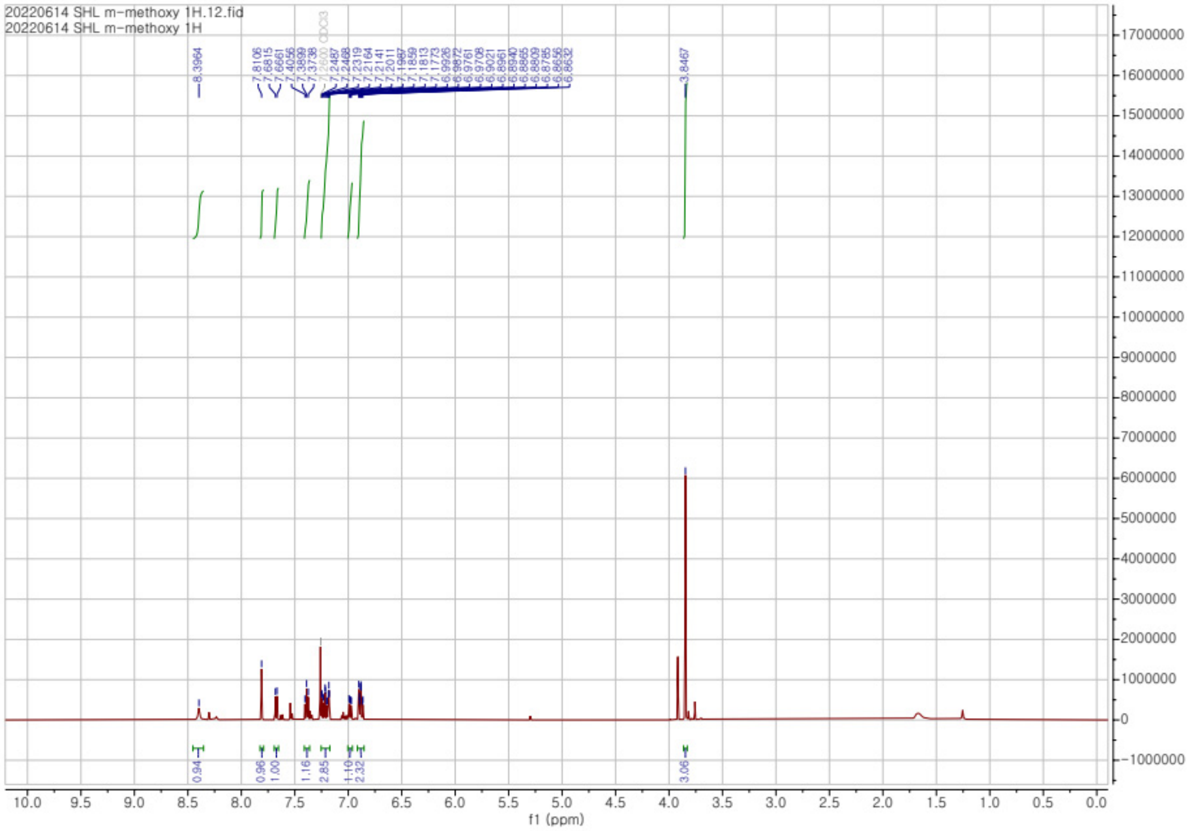

13C NMR of (Z)-3-(3-methoxybenzylidene)indolin-2-one

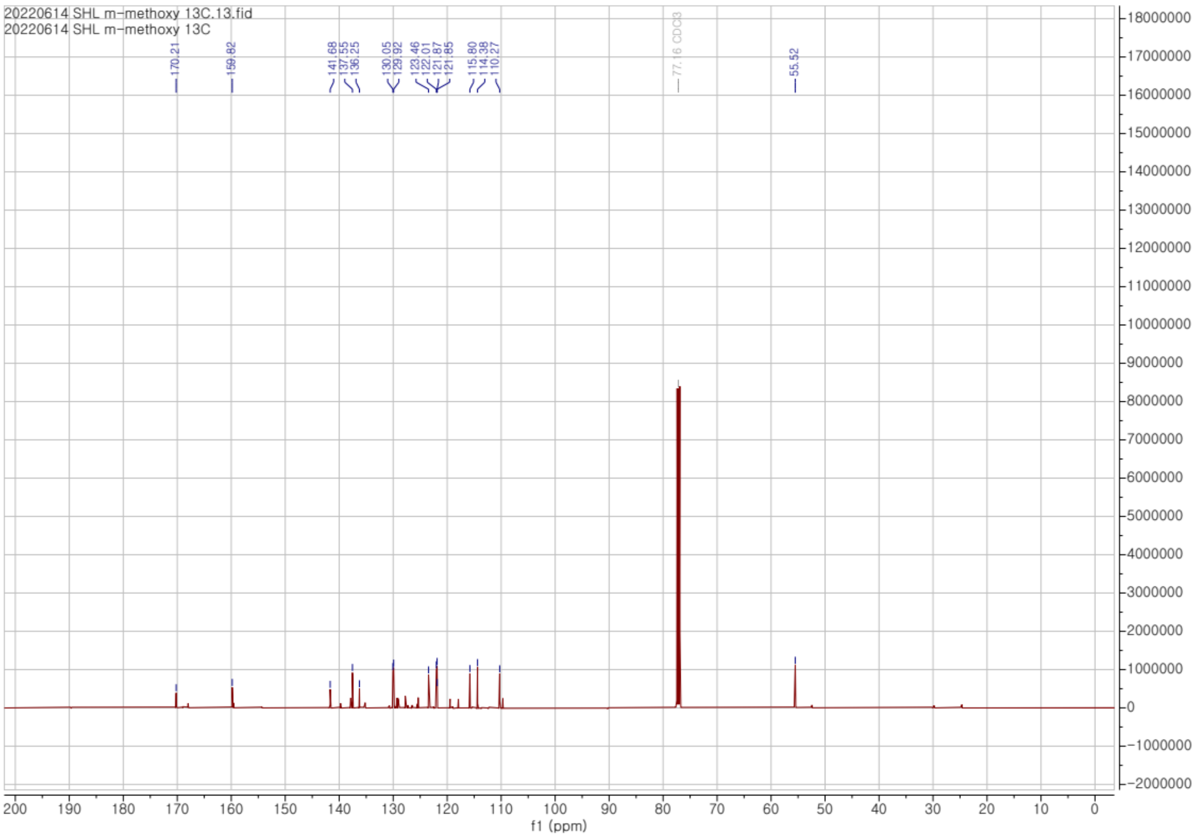

1H NMR of (Z)-3-(4-methoxybenzylidene)indolin-2-one

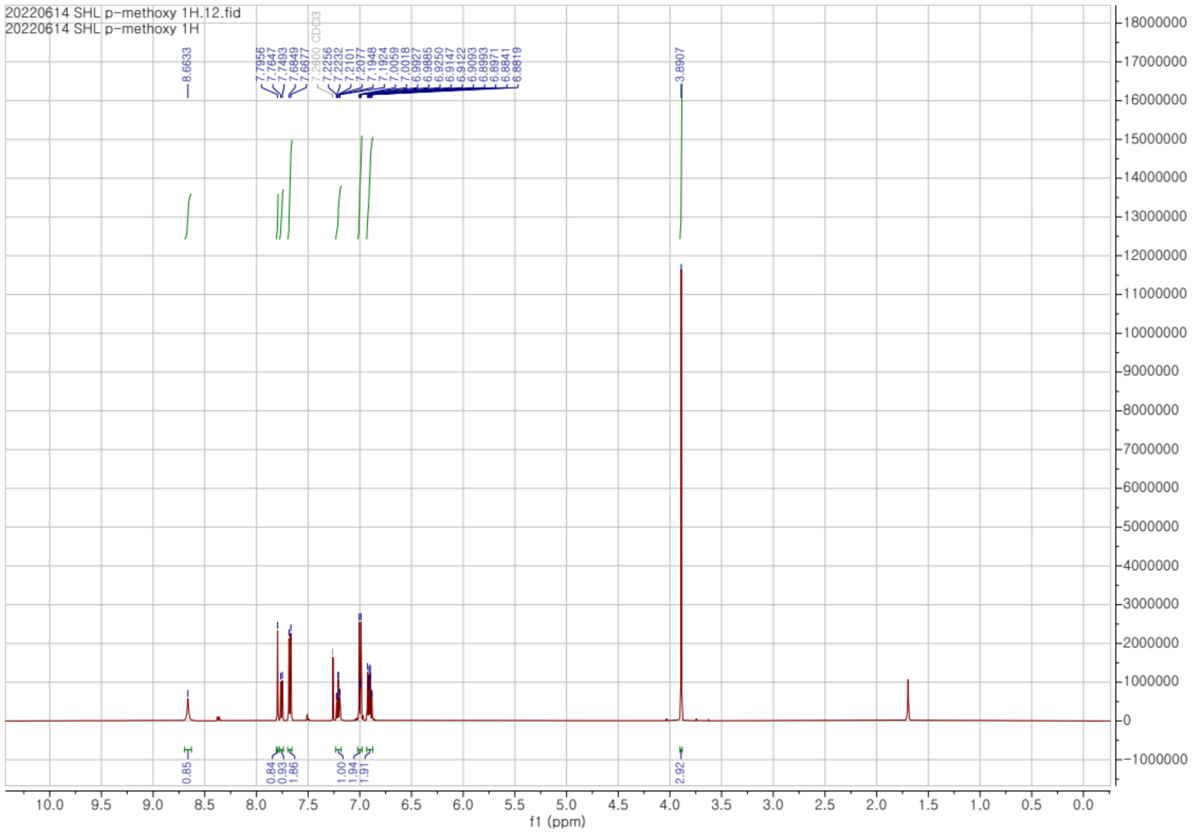

13C NMR of (Z)-3-(4-methoxybenzylidene)indolin-2-one

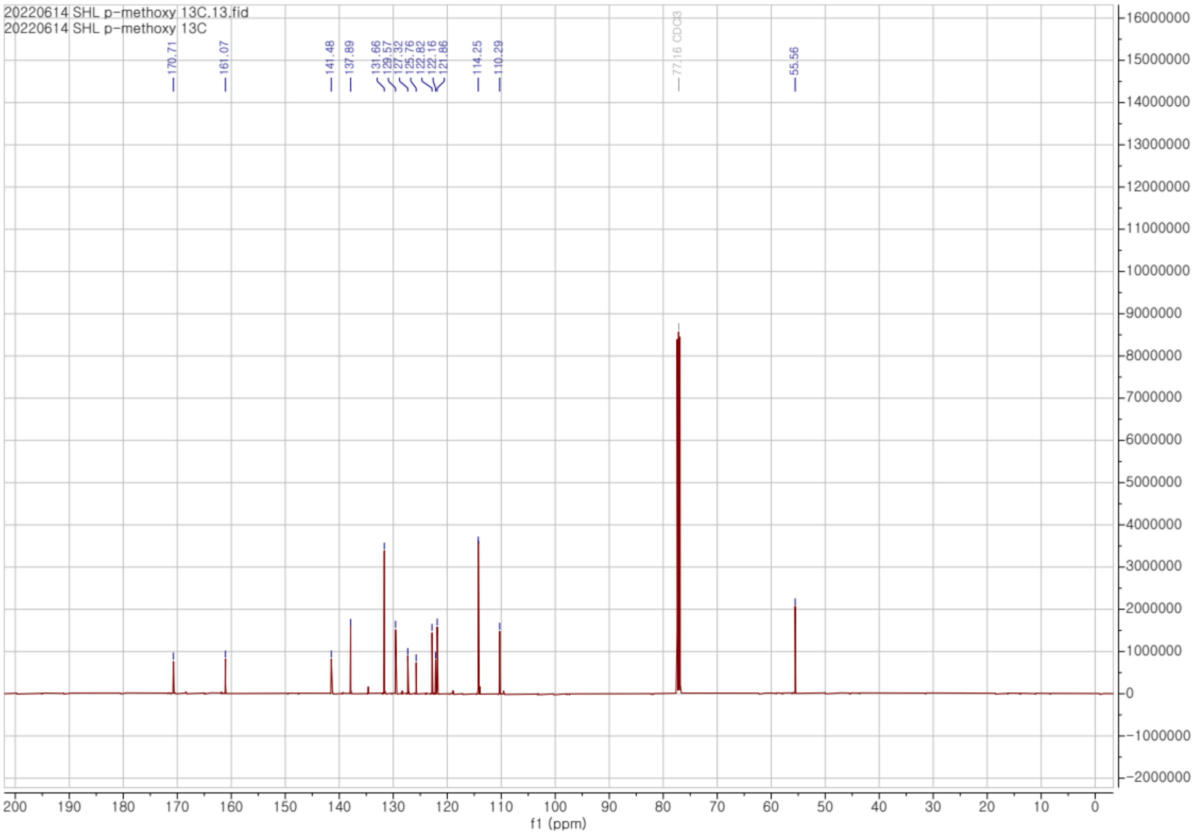

20220614 SHL p-methoxy 13C.13.fid
20220614 SHL p-methoxy 13C
170.71
161.07
141.48
137.89
131.66
129.57
127.32
125.76
122.82
122.16
121.86
114.25
110.29
77.16 CDCl3
55.56
f1 (ppm)
